# Collective Effervescence, Self-Transcendence, and Gender Differences in Social Well-Being During 8 March Demonstrations

**DOI:** 10.3389/fpsyg.2020.607538

**Published:** 2020-12-11

**Authors:** Larraitz N. Zumeta, Pablo Castro-Abril, Lander Méndez, José J. Pizarro, Anna Włodarczyk, Nekane Basabe, Ginés Navarro-Carrillo, Sonia Padoan-De Luca, Silvia da Costa, Itziar Alonso-Arbiol, Bárbara Torres-Gómez, Huseyin Cakal, Gisela Delfino, Elza M. Techio, Carolina Alzugaray, Marian Bilbao, Loreto Villagrán, Wilson López-López, José Ignacio Ruiz-Pérez, Cynthia C. Cedeño, Carlos Reyes-Valenzuela, Laura Alfaro-Beracoechea, Carlos Contreras-Ibáñez, Manuel Leonardo Ibarra, Hiram Reyes-Sosa, Rosa María Cueto, Catarina L. Carvalho, Isabel R. Pinto

**Affiliations:** ^1^Department of Social Psychology, Faculty of Psychology, University of the Basque Country UPV/EHU, San Sebastian/Donostia, Spain; ^2^School of Psychology, Catholic University of North, Antofagasta, Chile; ^3^Department of Psychology, Faculty of Humanities and Education Sciences, University of Jaén, Jaén, Spain; ^4^Department of Clinical and Health Psychology and Research Methods, Faculty of Psychology, University of the Basque Country UPV/EHU (for its Spanish/Basque initials), San Sebastian/Donostia, Spain; ^5^School of Psychology, Keele University, Staffordshire, United Kingdom; ^6^Centre of Research in Psychology and Psychopedagogy, Pontifical Catholic University of Argentina, Buenos Aires, Argentina; ^7^Laboratory for the Study of Psychological and Social Processes (LEPPS), Institute of Psychology, Federal University of Bahia, Salvador de Bahía, Brazil; ^8^School of Psychology, Santo Tomas University, Concepción, Chile; ^9^Faculty of Psychology, Alberto Hurtado University, Santiago, Chile; ^10^Department of Psychology, Faculty of Social Sciences, Concepcion University, Concepción, Chile; ^11^Department of Psychology, Pontifical Xavierian University, Bogota, Colombia; ^12^Department of Psychology, National University of Colombia, Bogota, Colombia; ^13^Faculty of Psychology, Salesian Polytechnic University, Quito, Ecuador; ^14^Andean Human Rights Program, Andean Simón Bolivar University, Quito, Ecuador; ^15^Department of Communication and Psychology, University Centre of Ciénega, University of Guadalajara, Ocotlán, Mexico; ^16^Laboratory of Social Cognition, Department of Sociology, Autonomous Metropolitan University, Iztapalapa, Mexico; ^17^University Campus in Nezahualcóyotl, Autonomous University of the State of Mexico, Nezahualcóyotl, Mexico; ^18^Department of Social Psychology, Autonomous University of Coahuila, Saltillo, Mexico; ^19^Department of Psychology, Pontifical Catholic University of Peru, Lima, Peru; ^20^Laboratory of Social Psychology, Faculty of Psychology and Education Sciences, University of Porto, Porto, Portugal

**Keywords:** collective effervescence, perceived emotional synchrony, 8M demonstrations, self-transcendence, well-being, participation in collective rituals, feminist demonstrations, gender differences

## Abstract

8 March (8M), now known as International Women’s Day, is a day for feminist claims where demonstrations are organized in over 150 countries, with the participation of millions of women all around the world. These demonstrations can be viewed as collective rituals and thus focus attention on the processes that facilitate different psychosocial effects. This work aims to explore the mechanisms (i.e., behavioral and attentional synchrony, perceived emotional synchrony, and positive and transcendent emotions) involved in participation in the demonstrations of 8 March 2020, collective and ritualized feminist actions, and their correlates associated with personal well-being (i.e., affective well-being and beliefs of personal growth) and collective well-being (i.e., social integration variables: situated identity, solidarity and fusion), collective efficacy and collective growth, and behavioral intention to support the fight for women’s rights. To this end, a cross-cultural study was conducted with the participation of 2,854 people (age 18–79; *M* = 30.55; *SD* = 11.66) from countries in Latin America (Mexico, Chile, Argentina, Brazil, Peru, Colombia, and Ecuador) and Europe (Spain and Portugal), with a retrospective correlational cross-sectional design and a convenience sample. Participants were divided between demonstration participants (*n* = 1,271; 94.0% female) and non-demonstrators or followers who monitored participants through the media and social networks (*n* = 1,583; 75.87% female). Compared with non-demonstrators and with males, female and non-binary gender respondents had greater scores in mechanisms and criterion variables. Further random-effects model meta-analyses revealed that the perceived emotional synchrony was consistently associated with more proximal mechanisms, as well as with criterion variables. Finally, sequential moderation analyses showed that proposed mechanisms successfully mediated the effects of participation on every criterion variable. These results indicate that participation in 8M marches and demonstrations can be analyzed through the literature on collective rituals. As such, collective participation implies positive outcomes both individually and collectively, which are further reinforced through key psychological mechanisms, in line with a Durkheimian approach to collective rituals.

## Introduction

In this work, we aim to study the relationship between participation in the 8 March (8M) demonstrations and personal and collective well-being and to explore the psychosocial mechanisms involved in this relationship. 8M, now known as International Women’s Day, is a date to commemorate the long history of struggle and sacrifice to obtain women’s rights. While there is debate between multiple versions claiming historic origins, the most popular one is associated with working-class women’s demonstrations, the Suffragist Movement (Castaño-Sanabria, 2016), and/or the tragic fire at a textile factory in New York in 1911, where more than 100 women employees perished (Ortega, 2019). However, the most plausible interpretation of 8M’s historic origins falls under the socialist movement claiming labor rights (Percy, 2014; Awcock, 2020).

Despite many years of fighting for women’s rights, acknowledgment of equality among all human beings at the Human Rights Convention of 1945, the International Bill of Human Rights for Women of 1979, and multiple conventions (e.g., Fourt World Conference on Women in Beijing, 1995, see United Nations [UN], 1996) and legislations seeking to tackle inequality between men and women over the past 75 years, persistent gender-based discrimination can be easily identified almost everywhere in the world. Numerous data and research studies confirm that, still in the twenty-first century, being a woman is a social burden with consequences in all areas (e.g., World Economic Forum [WEF], 2020).

For all these reasons, 8M is, par excellence, the day for feminist claims, organizing marches and demonstrations in over 150 countries, with the participation of millions of women (along with some men and other people of non-binary identities). It draws noteworthy social and media visibility all around the world (e.g., Franco, 2018), as it states its nonconformity with the patriarchal structure that discriminates against women. Feminism today, a social movement with a long, extensive, diverse and globalized, transnational and intersectional history, is difficult to define based on one sole focus, since different ideological factions and very different socio-structural realities co-exist within the social movement (e.g., García Jiménez et al., 2016; Pellicer and Asin, 2018; Curiel, 2019). In line with other research, this study is based on the premise that feminism must be understood as a social movement based on the belief that women and men are equal and must have the same rights, and whose ultimate objective is to put an end to the subordination of women (Basow, 1992; Pellicer and Asin, 2018).

Additionally, demonstrations are a public and collective display of a collective’s opposition to or dissatisfaction with policies and practices of institutions and governments; as such, it is a customary and relevant tactic in all social movements (Tarrow, 2011). Previous research on activism and collective action have has that collective participation is an essential source of well-being (Klar and Kasser, 2009; Boffi et al., 2016; Hopkins et al., 2016), providing feelings of connection, feeling of community, and increased perception of social support. This, in turn, has been proven to have a substantial impact on psychological well-being (e.g., Berkman et al., 2000; Townley et al., 2011), especially for disadvantaged groups (e.g., Finch and Vega, 2003; Noh and Kaspar, 2003).

In this work, we study the relationship between participation in the 8M demonstrations, affective well-being, the connection with values and beliefs, social cohesion and integration, individual and collective empowerment, and the intention of prosocial behavior focused on the struggle for women’s rights. First, we propose that 8M demonstrations, with a long tradition of annual periodicity (fixed and pre-established date), stereotyped synchronic behavior or gestures (e.g., the raised hands building a triangle, dances), consolidated common symbols (purple color, iconography, and identification symbols), and shared values, can be partially conceived of as collective rituals (Collins, 2004; Watson-Jones and Legare, 2016), considering that they are a “mechanism of mutually focused emotion and attention producing a momentarily shared reality, which thereby generates solidarity and symbols of group membership” (Collins, 2004, p. 7). Moreover, collective rituals are symbolic, repetitive, and stereotyped behaviors that occur within a specific space-and-time frame (Páez et al., 2015). They foment shared meaning aimed at building a sense of community, social solidarity, and conformity with group values (Collins, 2004; Durkheim, 1912/2008). They provide a sense of community and connection, and high social and emotional interaction in addition to opportunities for citizen participation and shared meaning (e.g., Berkman et al., 2000; Hobson et al., 2018). However, we must not fail to mention that demonstrations have a certain degree of spontaneity; their rules are not rigid, and they have instrumental objectives, such as demanding or supporting legislative changes, which are the objectives of social movements (Basabe et al., 2004; Tarrow, 2011).

Consequently, we propose that the 8M demonstrations in favor of women’s rights as collective rituals be characterized by increased social interaction, a shared meaning intended to create a feeling of community (de Rivera and Mahoney, 2018), and social solidarity based on a shared objective. While it has been demonstrated that participation in collective rituals or collective ritualized actions improves personal well-being (e.g., Tewari et al., 2012; Páez et al., 2015) and collective well-being (e.g., Zumeta et al., 2016), surprisingly, we find no previous work that has focused on studying the 8M demonstrations from this perspective.

With this research, we shall examine different psychosocial mechanisms involved in this relationship, integrating different theoretical perspectives: rituals and collective effervescence (e.g., Durkheim, 1912/2008; Collins, 2004; Páez et al., 2015), positive psychology (e.g., Fredrickson, 2009; Zickfeld et al., 2019), including relevant aspects of collective-action theory (e.g., van Zomeren et al., 2008), and the social-identity approach (Tajfel and Turner, 1979; Drury and Reicher, 2009; Novelli et al., 2013). An initial mechanism during the process of emotional connection must necessarily be cognitive-behavioral; the behavioral and attentional synchrony, meaning synchronized behavior (e.g., frequency, rhythm, and movement), as well as shared and focused attention, can promote shared emotions, and act as forerunners to collective effervescence (Wlodarczyk et al., 2020, in this monograph). Another mechanism that is essentially affective is emotional effervescence or perceived emotional synchrony (PES), a feeling of convergence and alignment in emotional responses that occurs among participants at a collective meeting (Durkheim, 1912/2008; Páez et al., 2015; Xygalatas et al., 2016). A third mechanism is the intense concurrent positive emotional experience (e.g., Fredrickson, 2009), and the fourth are positive self-transcendent emotions, meaning emotions that project our being outward and promote connection with others (e.g., Haidt, 2003; Stellar et al., 2017) in the context of social interaction.

Finally, based on the theory of collective action and social change (van Zomeren et al., 2008), we posit that participation in the collective action intended to reduce injustice/inequality and to change the status quo (Dixon et al., 2017) can have positive effects on well-being and empowerment, both personally (beliefs of individual growth) and as a collective, increasing group efficacy (Ohmer, 2010; Carbone and McMillin, 2019; Zabala et al., 2020, in this monograph) and positive collective growth (McNamara et al., 2013; Wlodarczyk et al., 2016; Bilali et al., 2017).

### Participation in 8 March Demonstration, Antecedents of Perceived Emotional Synchrony, and Collective Effervescence

Collins (2004) states that co-presence and shared attention as the result of participating in collective rituals create a shared reality and reinforce inter-subjectivity, creating effervescence through shared ideas and emotions. This is based on Émile Durkheim’s concept of collective effervescence, which includes attentional convergence (i.e., shared focus of attention) and behavioral and expressive synchrony (coordination of movements and gestures), and, especially, emotional synchrony (coordination and convergence of all emotional facets). After synchronizing and coordinating their attention and behavior, participants also synchronize their emotions, feeling something intense and similar that grows more intense due to mutual feedback (Durkheim, 1912/2008). PES is therefore an emotional experience had by participants during collective meetings. It represents the experience and feeling of bonding with others (Páez et al., 2015; Rimé et al., unpublished). In this regard, PES is successor to the notion raised by Durkheim (1912/2008) in the classic concept of collective effervescence, the intense shared emotional experience. PES implies a feeling of convergence and alignment of emotional responses that take place between participants in a collective meeting (Xygalatas et al., 2016; Rimé et al., unpublished). This is the effect of perception of similarity, convergence, and intensification of emotional evaluations, corporal and affective reactions, a subjective feeling, and action tendencies (see Rimé et al., unpublished). PES shows the emotional feeling of bonding that participants in 8M may experience in feeling bonded with the others in terms of affect, thought, and, more often, physical action and movement.

In this regard, we postulate that participation in the 8M demonstration causes collective effervescence, which provides communion or fusion of all feelings in a shared affective experience, and sharing emotions intensifies said affective experience. PES is the result not only of the experience of shared emotions felt but also of the entire experience of collective synchronization of all different facets of the affective experience.

#### Perceived Emotional Synchrony and Positive Emotions: Self-Transcendence and Self-Reference

PES is the joy of demonstrating and increased emotions of high excitement with clearly positive components (joy and euphoria) among those participating in a successful collective meeting, whether a demonstration, a ritual, or any other kind of meeting (Moscovici, 1988; Hopkins et al., 2016). However, the affective content of the PES experience (meaning the intense emotion that is shared) may vary depending on the specificity of the collective situation occurring (Páez et al., 2015). For example, with 8M demonstrations, moral indignation, hope, and the joy of participating may prevail, while in other contexts, the dominant emotional content may imply, for example, pride in a religious or secular ritual of glorification (e.g., Draper, 2014; Sullivan, 2014; Hopkins et al., 2016); and in a more negative context, fear and rage might prevail, as occurs in certain political rituals, or even pain, sadness, and guilt, as occurs in certain religious rituals (Sullivan, 2014).

Moreover, we can expect that collective effervescence will be related to the emotional experience of self-transcendence. It has been proven that a subset of positive emotions (sometimes called “emotions of self-transcendence,” “moral emotions,” or “other positive emotions of worship”) are able to mobilize people to connect with others in their environment or society. These emotions are elevation or moral inspiration, compassion, gratitude, feeling moved by love, and wondrous awe when witnessing a grandiose social object (Haidt, 2003; Algoe and Haidt, 2009; van Cappellen and Rimé, 2014). These are emotions sparked by assessments focused on others, based on shifting attention toward the needs and concerns of others (for example, suffering, virtue, moral inspiration and awe, and love and being close to others), so they decrease the prominence of the individual self and promote bonding with other people and social groups (Haidt, 2003; van Cappellen and Rimé, 2014; Stellar et al., 2017). They are related to the interests or well-being of either society as a whole or, at least, the people who are neither the judge nor agent (Haidt, 2003). As a result, they constitute powerful determining factors in prosocial behavior or behavior to help others (Goetz et al., 2010; Cusi et al., 2018; Pizarro et al., 2018). As such, they are in clear contrast with positive emotions that are the result of self-referential assessments (focused on the self) as occurs when the self has experienced a positive emotion (joy) or great success (pride).

### Participation in Demonstrations as Social Belonging and Well-Being

Previous studies have shown that PES is associated with social identification, social integration, fusion identity, enhanced personal and collective empowerment, positive affect, and positive shared beliefs among participants (Collins, 2004; Páez et al., 2015; Wlodarczyk et al., 2020). These results were similar for positive valence events (folk celebrations) and mixed- or negative-valence events (Páez et al., 2015; Zumeta et al., 2016; Wlodarczyk et al., 2020). The present study focuses on the social and individual effects of collective feminist demonstrations. Therefore, we argue that participation in these collective gatherings will enhance identification with feminist organizations, foster collective efficacy and growth in the aftermath of the demonstrations, and finally, will increase prosocial behaviors. Additionally, we will pay attention to the role played by emotional bonding in the way that such effects occur.

First, it has been shown that participation in collective emotional gatherings increases identification with other co-present participants and also reinforces a broader sense of social identity (i.e., ethnic identification; Gasparre et al., 2010; Khan et al., 2015) and enhances prosocial behaviors (Rossano, 2012). Furthermore, it has been suggested that rituals and collective gatherings may “fuel” identity fusion with other members of the group (Swann et al., 2012). Identity fusion, or blurring of the self-others boundary between the personal and collective self, encourages people to channel their personal agency into group behavior, motivating pro-group behavior, both aggressive and altruistic, and is related to well-being (Gómez et al., 2011; Swann et al., 2012; Zabala et al., 2020, in this monograph). Second, participation in collective emotional gatherings enhances different facets of social belonging, such as social integration (Weiss and Richard, 1997) and perception of social support (Páez et al., 2007), and increases social cohesion by reinforcing positive inter-group stereotypes (Kanyangara et al., 2007), which reinforces a positive emotional climate (de Rivera and Páez, 2007; Pelletier, 2018; Bouchat et al., 2020; Rimé et al., unpublished) and predicts solidarity (Hawdon and Ryan, 2011). Third, participation elicits positive individual emotions (Neville and Reicher, 2011) and collective emotions (Páez et al., 2007, 2013) and predicts increases in well-being (Tewari et al., 2012). Fourth, it empowers participants and consequently increases their personal and collective sense of efficacy, self-esteem, and post-stress growth (Drury and Reicher, 2005; Páez et al., 2007; Rimé et al., unpublished; Zabala et al., 2020, in this monograph; Zumeta et al., 2016). Finally, collective gatherings reinforce agreement with “sacred” symbols and values (Collins, 2004; Páez et al., 2007; Fischer et al., 2014; Gabriel et al., 2020).

These effects would be explained by the PES and the emotions experienced at the demonstration. In this sense, 8M demonstrations in comparison with non-demonstrations will report not only higher well-being but also higher perceived attentional and behavioral synchrony, PES, more positive and transcendent emotions, and more agreement with the values promoted by the movement. Following Páez et al. (2015) and Wlodarczyk et al. (2020, in this monograph), we propose and will contrast a sequential model. First, participation in demonstrations affords attentional and behavioral synchrony (and mass and social media facilitate perception of said demonstration). Second, attentional and behavioral synchrony, along with bottom-up processes such as expressive and verbal affect-loaded behavior, and top-down process such as shared appraisals of issues, goals, and values, elicit collective effervescence or shared, convergent, and coordinated emotional responses (i.e., PES). Even if shared, convergent and coordinated emotional states could be negative or ambivalent; emotional synchrony in general intensifies emotions and fuels the “joy of being together” or intense positive emotions as the initial consequences of PES. Moreover, because collective gatherings and rituals connect people with large categories and social goals, emotional synchrony during demonstrations and ceremonies elicits positive self-transcendent emotions as a second consequence. Finally, because collective gatherings are loaded-value, emotional synchrony and intensification of positive and self-transcendent emotions are conducive to “contact with the sacred” or salience and adhesion to cultural values (see Wlodarczyk et al., 2020, in this monograph for the discussion of antecedents, content of emotional synchrony, and proximal and distal effects). Additionally, we expect that these effects will be more pronounced among women, who are the target or central category of the theme of the social movement in question.

In sum, if 8M demonstrations are ritualized forms of collective participation, and if they evoke a feeling of PES and intense emotions due to this, we could expect that demonstrators, compared with individuals who are non-participants but followers, will experience more PES and more positive and self-transcendent emotions and will manifest greater social cohesion (social identity, identity of fusion, and solidarity) and more agreement with the values promoted by the ritualized collective action.

This hypothesis is consistent with a previous and continuous line of research about participation and the role of PES (collective effervescence measure) as a predictor or mediator of the positive causal effects of participation in rituals and collective gatherings (derived from the theoretical tradition of Durkheim, 1912/2008; Collins, 2004). It must be noted that previous longitudinal studies (pretest-during-posttest) have shown that PES predicts the positive effects of participation (e.g., von Scheve et al., 2017; Bouchat et al., 2020; Pizarro et al., 2020; Wlodarczyk et al., 2020; Zabala et al., 2020).

### Objectives and Hypotheses

The objective of this work is to explore the psychosocial mechanisms (behavioral and attentional synchrony, PES, and positive and self-transcendent emotions) involved in participation in demonstrations on 8 March 2020, collective and ritualized feminist actions, and their psychosocial correlates. These correlates are affective well-being, connection with values and beliefs, social well-being based on cohesion and social integration (situated social identity, identity fusion with demonstrators and feminists, in-group solidarity and identity fusion with women), empowerment (collective efficacy and beliefs in individual and collective growth), and the intention of prosocial behavior aimed toward the fight for the rights of women in different countries.

**FIGURE 1:**
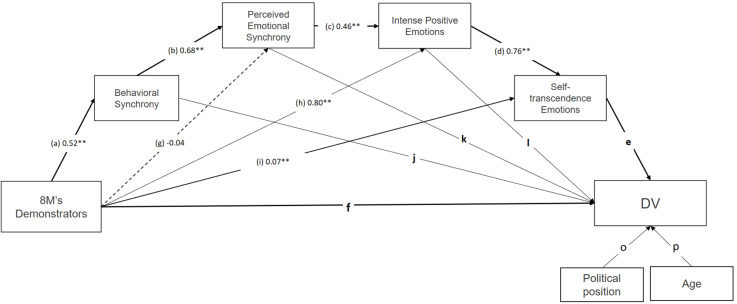
Model of multiple serial mediation with female sample. Note. Standardized direct effects were reported. ^∗∗^*p* < 0.01.

To this end, we studied participation in 8M demonstrations. First, we verified the differences between the demonstrators and the non-demonstrators (audience or mass-media followers), as well as differences based on gender, using mean comparison. Moreover, we calculated average effect sizes with random effects for the countries total. Finally, by using a sequential measurement model, we examined the mediator effect (indirect effects) of each one of the psychosocial mechanisms, including PES in relation to each criterion variable. The criterion variables were as follows: the experience of transcendence, the situated social identity, identity fusion (demonstrators, feminists, and women), solidarity, collective efficacy, individual and collective positive growth, and, lastly, the intention to help fight for women’s rights. Indirect effects of age and political-position variables were controlled out of all variables in the model. The sequential mediation model set forth above (Goetz et al., 2010; Cusi et al., 2018; Pizarro et al., 2018) is shown in Figure 1.

Accordingly, the following hypotheses were raised: (H1) During the 8M, behavioral and attentional synchrony will occur, along with emotional synchrony (PES-collective effervescence); moreover, positive emotions will be activated with high intensity, including emotions of transcendence, both in demonstrators and in non-demonstrators and followers alike. (H2) The psychosocial mechanisms will be linked to the effects: the experience of self-transcendence, situated social identity, and identity fusion with collectives representing the ritual (marchers, feminists, and women), solidarity with women, collective efficacy, individual and collective growth, and the intention of behavior linked to the movement for women’s rights (Páez et al., 2015; Zumeta et al., 2016; Rimé et al., unpublished; Wlodarczyk et al., 2020). (H3) The demonstrators and women (in comparison with non-demonstrators and men) will display higher scores both in the psychosocial mechanisms involved and in criterion variables, due to the effects of emotional activation from participating and from women being the central identity of the demonstration and social movement. (H4) Participation in 8M demonstrations will increase in accordance with values, personal well-being (positive individual growth), and collective or social well-being, including aspects of cohesion and social integration (social identity, identity fusion, and solidarity with women), and of collective empowerment (collective efficacy, positive collective growth) and the intention of helping behavior (Basabe et al., 2004; Albanesi et al., 2007). This will be mediated by PES antecedents (attentional and behavioral synchrony), PES, the intense positive emotions, and emotions of self-transcendence (see Figure 1).

## Materials and Methods

### Participants

A cross-sectional, correlational study design was used. A cross-cultural study is provided, including samples from Latin America (Mexico, Chile, Argentina, Brazil, Peru, Colombia, and Ecuador) and Europe (Spain and Portugal). In all nine countries, the 8M demonstrations showed similar characteristics. Symbolic elements were shared, as well as the use of choreography and dance, and a common language in favor of women’s rights. A brief ethnographic description is presented in Supplementary Table 7. The age range of participants is from 18 to 79 years, with 44.50% having attended the marches or demonstrations, as opposed to non-demonstrators^1^, audience, or followers on 8M. In both groups, the female percentage who responded to the survey was a majority, even more so and especially among the group of demonstrators (see Table 1).

**TABLE 1:** Demographic characteristics of sample.

Characteristics	Full sample	Demonstrators	Non-demonstrators
**Age: *M* (*SD*)**	30.55 (11.66)	32.04 (11.88)	29.35 (11.34)
**Gender**			
Female	83.8%	94.0%	75.9%
Male	14.8%	4.1%	23.4%
Non-binary	1.3%	1.9%	0.9%
**Education**			
High	86.4%	89.0%	84.4%
Low	13.6%	11.0%	15.6%
**Political positioning**			
Left	65.8%	85.6%	47.3%
Center	23.3%	13.9%	32.0%
Right	1.4%	0.5%	2.3%
No positioning	9.5%	0%	18.5%
***N***	2,854	1,271	1,583

*N = 2,854. Valid percentage (%) is reported. Education: dichotomized based on four levels (high = university, low = primary, secondary, tertiary). Political positioning: continuous categorized scale 1–7 (left = 1 and 2, middle = 3, 4, and 5, and right = 6 and 7).*

Table 2 displays descriptive analysis, including mean and standard deviation for age and frequency distribution, by gender and participation in the 8M demonstrations for each country. As shown in the table provided, the proportion of female respondents in all countries was substantially higher than that of male and non-binary individuals. Specifically, the proportion of females ranged from 75.5% in the case of Peru to 88.9% in Brazil. Regarding the level of participation in the 8M demonstrations, it fluctuates from 12.1% in the Colombian sample to 74.9% in the Chilean sample. The proportion of respondents that participated in the 8M demonstrations was higher than 50% in four of the nine countries assessed [i.e., Chile (74.9%), Portugal (62.7%), Ecuador (56.3%), and Spain (51.4%)]. For more information, see Supplementary Table 1.

**TABLE 2:** Descriptive analysis for each country sample.

Country	*N*	Age *M*	(*SD*)	% Female	% Demonstrations	Continent
Argentina	207	22.0	5.3	87.0	24.2	Latin America
Brazil	72	35.5	14.8	88.9	23.6	Latin America
Chile	475	29.7	11.3	86.5	74.9	Latin America
Colombia	190	23.6	9.8	75.3	12.1	Latin America
Ecuador	103	34.6	10.2	78.6	56.3	Latin America
Spain	457	37.0	14.2	84.0	51.4	Europe
Mexico	1,032	30.1	9.4	86.0	39.7	Latin America
Peru	245	29.8	11.9	75.5	32.2	Latin America
Portugal	67	33.4	13.9	77.6	62.7	Europe

*N = 2,843 (11 subjects eliminated for not reporting their country).*

### Procedure

Contact was established with social psychology research groups in Latin America and in Europe for a cross-cultural sample. With the Qualtrics Survey Platform®, online surveys were prepared both in Spanish and in Portuguese, accessed via a link. After the demonstrations on 8M, the links were shared with those who participated directly in the 8M 2020 demonstrations (demonstrators) and people who had followed the demonstrations through mass media and social networks (non-demonstrators, audience, and followers). The data were collected between 8 March and 30 March^2^ 2020 in nine different countries, and the approximate time spent on the survey was 30 min. The sample collected was convenience sample.

The procedure for data collection in all countries was similar; convenience sampling was used in all locations, and QR codes from the Qualtrics application were shared through a snowball scheme (see Supplementary Table 8). The sample differences between countries are mostly related to the number of collaborating research groups in each country. In Argentina, Chile, and Spain, the samples are larger because two or more groups were involved in the sample collection.

All study participants read and accepted informed consent. The data recorded were alphanumerically code to ensure anonymity following the Organic Law on the Protection of Personal Data (BOE-A-2018-16673) and compliance with the regulation of the Ethics Committee for Research Involving Human Beings (CEISH) by the University of the Basque Country.

### Measurements

The scales used in this research are based on a proposal made by Wlodarczyk et al. (2020, on this issue) for an integrative measurement of collective effervescence experiences. The Supplementary Material includes confirmatory factor analysis (CFA) for each instrument showing appropriate adjustment rates for the one-dimensional structure at all scales (Table 2) and a reliability analysis (Cronbach’s α) for each instrument by country (Table 3).

**TABLE 3:** Descriptive analysis and correlations among target variables.

	Variables	1	2	3	4	5	6	7	8	9	10	11	12	13	14	15	16	17	18
1	Participation	1																	
2	Behavioral synchrony	0.236**	1																
3	Perceived emotional synchrony	0.140**	0.690**	1															
4	Intense positive emotions	0.467**	0.532**	0.608**	1														
5	Self-transcendent emotions	0.416**	0.548**	0.656**	0.901**	1													
6	Self-transcendent experience	0.359**	0.575**	0.692**	0.787**	0.837**	1												
7	Situated social identity	0.438**	0.526**	0.622**	0.821**	0.847**	0.823**	1											
8	Identity fusion demonstration’s	0.441**	0.407**	0.467**	0.671**	0.677**	0.645**	0.716**	1										
9	Identity fusion feminist’s	0.427**	0.359**	0.423**	0.643**	0.635**	0.587**	0.677**	0.716**	1									
10	Solidarity with women	0.297**	0.405**	0.452**	0.578**	0.619**	0.589**	0.639**	0.541**	0.566**	1								
11	Identity fusion women	0.125**	0.187**	0.232**	0.291**	0.312**	0.288**	0.329**	0.402**	0.457**	0.436**	1							
12	Collective efficacy	0.241**	0.365**	0.421**	0.535**	0.587**	0.555**	0.540**	0.459**	0.461**	0.571**	0.272**	1						
13	Positive individual growth	0.265**	0.365**	0.446**	0.596**	0.644**	0.644**	0.638**	0.510**	0.448**	0.474**	0.268**	0.473**	1					
14	Positive collective growth	0.329**	0.414**	0.512**	0.661**	0.699**	0.688**	0.676**	0.556**	0.524**	0.537**	0.259**	0.580**	0.699**	1				
15	Pro-women behavior	0.439**	0.395**	0.454**	0.683**	0.696**	0.673**	0.718**	0.608**	0.631**	0.565**	0.268**	0.516**	0.591**	0.608**	1			
16	Political orientation	0.234**	0.055**	0.013	0.009	–0.019	0.011	–0.008	0.027	0.014	0.003	–0.006	–0.036	–0.035	–0.011	–0.037	1		
17	Gender	−0.208**	−0.107**	−0.143**	−0.206**	−0.204**	−0.182**	−0.212**	−0.216**	−0.214**	−0.135**	−0.206**	−0.091**	−0.157**	−0.176**	−0.205**	0.007	1	
18	Age	0.115**	−0.065**	−0.101**	–0.015	–0.030	−0.071**	0.017	–0.005	–0.036	0.021	–0.009	−0.050**	−0.098**	−0.064**	−0.047*	0.018	0.005	1
	*M*	–	5.18	5.58	5.00	5.32	5.18	5.01	3.46	3.53	6.10	4.03	5.45	4.03	4.64	3.46	2.20	–	30.55
	*SD*	–	1.47	1.49	1.90	1.81	1.75	1.96	1.32	1.34	1.28	1.00	1.38	1.65	1.42	1.22	1.70	–	11.66
	Range	0–1	1–7	1–7	1–7	1–7	1–7	1–7	1–5	1–5	1–7	1–5	1–7	0–5	0–5	1–5	0–7	1–2	18–
	Items	1	2	6	3	5	4	3	1	1	3	1	4	3	3	5	1	1	79
	*N*	2,854	2,854	2,854	2,854	2,854	2,854	2,854	2,832	2,832	2,752	2,832	2,854	2,752	2,752	2,752	2,669	2,854	2,849

Participants (0 = non-demonstrator/followers/audience, 1 = demonstrator), gender (1 = female, 2 = male, 3 = non-binary), and political position (0 = no position, 1 = extreme left to 7 = extreme right). *p ≤ 0.05; **p ≤ 0.001.

#### Mechanisms

##### Antecedents to Collective Effervescence: Shared Attention and Behavioral Synchrony

Based on Collins (2004), Rennung and Göritz (2016), and Gabriel et al. (2017), two *ad hoc* items were developed to measure the shared attention and behavioral synchrony, antecedents of collective effervescence (e.g., *the participants focused their attention on the same symbols, objects, and events*). A Likert scale was used, with a response range of 1 (*Totally disagree*) to 7 (*Totally agree*). The reliability coefficient was adequate (Cronbach’s α = 0.729).

##### Perceived Emotional Synchrony

A reduced version of six items was used (see Wlodarczyk et al., 2020) of the PES scale (Páez et al., 2015) to assess the degree of infection or sharing emotion experienced, and perception of emotional synchrony with the other co-participants (e.g., *We felt more intense emotions because we all went through the same experience*). Response ranges go from 1 (*Totally disagree*) to 7 (*Totally agree*). Cronbach’s coefficient was high (α = 0.883).

#### Proximal and Distal Effects

##### Positive Emotions

Two types of positive emotions were assessed. *Intense positive emotions*: Research was conducted on the prototypical positive emotions from the N (2013) scale with three items referring to feeling *realized*, *happy*, and *alive* during the 8M demonstration. The response range went from 1 (*Nothing*) to 7 (*Totally*). Cronbach’s reliability coefficient was high (α = 0.932). *Transcendent emotions*: Participants were asked about the transcendent emotions they felt in relation to the 8M demonstration, using the adapted version of the DES scale (based on Fredrickson, 2009; Zickfeld et al., 2019) with five Likert-style items (e.g., *In Awe*, *Amazed*, *Overwhelmed by something grand*, *Morally inspired*, and *Uplifted*). The response range went from 1 (*Nothing*) to 7 (*Totally*). Cronbach’s reliability coefficient was very high (α = 0.955).

#### Connection/Agreement With Values and Beliefs

##### Transcendent Experience

Four Likert-style items were used to research (e.g., *I felt like there was something transcendent, associated with values and ideals, above the action*) the degree of transcendence experienced by the subjects in relation to the 8M demonstration (Gabriel et al., 2020). Response ranges went from 1 (*Totally disagree*) to 7 (*Totally agree*). The reliability coefficient was very satisfactory (α = 0.922).

#### Social Cohesion and Social Integration

##### Situated Social Identity

Participants were asked about their degree of identification with the demonstrators (Novelli et al., 2013) by means of three items (e.g., *I identified with the demonstrators*). Response ranges went from 1 (*Totally disagree*) to 7 (*Totally agree*). The reliability coefficient score was very high (α = 0.946).

##### Pictorial Identity Fusion

To assess identity fusion, the pictorial scale of identity fusion was used (Gómez et al., 2011). Based on the measurement “Inclusion of other in the self (IOS) Scale” (Aron et al., 1992), this consisted of a pictorial item that shows the perception of closeness or fusion with a reference group. Three items were included (*Which picture best describes your relationship with …*), one for each reference group, two situated within the context (participants in the specific demonstration and feminists), and another one as a general category, with women (e.g., *all the women in the world?*). The five response options range from A to E, where A symbolizes a lesser perception of closeness or fusion (i.e., circles without overlapping) and E is a greater closeness or fusion (i.e., completely overlapping circles).

##### In-Group Solidarity

Three items with statements were used to assess solidarity and commitment to women [e.g., *I feel (morally) committed to women*], taken from Leach et al. (2008)’s Social Identity scale, with a response range of 1 (*Totally disagree*) to 7 (*Totally agree*). The reliability coefficient was satisfactory (α = 0.908). This version of the scale has been applied in different research works, demonstrating reliability and structural validity with a common dimension (Bobowik et al., 2013).

#### Empowerment

##### Collective Efficacy

Four items extracted from van Zomeren et al. (2010) were used in relation to perception of the efficacy of the reference group, in this case, the women (e.g., *I think that together with women and men, we can change the current situation*). The response range goes from 1 (*Totally disagree*) to 7 (*Totally agree*), and the reliability coefficient (Cronbach’s α) was high (α = 0.919).

##### Positive Growth

In order to assess positive growth, we used six items of positive growth scale from Páez (2011): three items for positive individual growth (e.g., *I have changed my priorities about what is important in life*), with a high reliability coefficient (α = 0.933), and three items for positive collective growth (e.g., *We have increased participation and political and ethical commitments for others*). The response range goes from 0 (*No change*) to 5 (*Very great*), with a reliability coefficient that is also high (α = 0.917).

#### Pro In-Group Behaviors

##### Pro-women Behavior

###### Intention of behavior to help women (ad hoc)

Seven Likert-style items were created to assess the participants’ behavior intention in future participation in actions, organizations, and initiatives for women’s rights (e.g., *Committing 2 h per week to collaborate with an association that organizes marches*). The response range goes from 1 (*Nothing*) to 5 (*A lot*). The reliability coefficient was high (Cronbach’s α = 0.890).

##### Sociodemographic Information

Participants provided information regarding their participation in 8M (0 = *non-demonstrator/followers/audience*, 1 = *demonstrator*) and their sociodemographic features: age, gender (1 = *female*, 2 = *male*, and 3 = *non-binary*), educational level (1 = *none or incomplete primary education*, 2 = *primary studies*, 3 = *lower and upper secondary education*, 4 = *first stage of tertiary education*, and 5 = *second stage of tertiary education*), and political position (1 = *extreme left* to 7 = *extreme right*, including the possibility of responding 0 = *no response or no position*).

### Design and Analyses

For this retrospective correlational cross-sectional and transnational research, we obtained descriptive statistics, reliability (Cronbach’s α) and correlations, and mean comparisons (GML) with SPSS® 26.0. To test indirect effects only on female participants, we used mediation analysis (Model 6), using the macro PROCESS 3.3 (Hayes, 2013). We used a bootstrapping estimation method based on 10,000 repetitions (Preacher and Hayes, 2004). The level of significance used was *p* < 0.05. We performed the confirmatory factorial analysis with JASP® 0.11 to verify the adequate adjustment to the one-dimensional theoretical structure of each scale.

In the analysis by countries, we applied meta-analytical techniques, following Cumming’s (2012) guides. We used Pearson’s *r*, calculated by countries, as the measurement of the size of the effect. We conducted a random-effects meta-analyses model. We explored the average effect size (magnitude) of the relationship between PES and each criterion variable, and heterogeneity indexes. To evaluate the effect sizes, the following criteria were adopted: *r* < 0.18 was small, 0.18 < *r* < 0.32 was medium, and *r* > 0.32 was large. This approach was undertaken due to the problematic use of Cohen’s (1977) rule of thumb (for further discussion, see Gignac and Szodorai, 2016; Funder and Ozer, 2019; Correll et al., 2020). Confidence intervals (CIs) of 95% and average effect size *r* are indicators of the validity of the magnitude of the effect or of the validity of the relation between the variables. Heterogeneity/homogeneity in effect sizes by country was calculated with the *Q* statistic. The following tests were also added: the Rosenthal test, fail-safe *N*, which reports the number of studies that must be added for the size of the average effect to be statistically insignificant, and Egger’s regression tests to detect possible publication or selection biases (see Rubio-Aparicio et al., 2018), all with Comprehensive Meta-Analysis 3.0 software (CMA; Borenstein et al., 2005).

## Results

### Descriptive and Correlational Analysis

Table 3 displays descriptive statistics (i.e., means and standard deviations) of each of the variables studied and the Pearson product-moment correlations between them. Missing values did not exceed 5% in any variable [except in political orientation (6.5%)]. All key variables show mean values above the midpoint of the scale (e.g., 3.50); the relatively high scores displayed by respondents on the measure of solidarity toward women should be underlined (*M* = 6.10, *SD* = 1.28). Furthermore, all variables of interest were positively and significantly associated with each other. The size of correlation coefficients indicates the presence of moderate-to-strong positive associations between the variables analyzed. The rank of correlations fluctuates from the lowest correlation obtained for identity fusion with women and behavioral synchrony (*r* = 0.187, *p* < 0.001) to the highest one found between intense positive emotions and self-transcendence emotions (*r* = 0.901, *p* < 0.001), confirming H1 and H2. For correlations *r* > 0.70, we tested the collinearity index (see Supplementary Table 2). All the values obtained are adequate [tolerance > 0.1, variance inflation factor (VIF) < 10; Rovai et al., 2013].

#### Differences Related to Participation and Gender in Criterion Variable

##### Mean Comparisons Between Demonstrators and Non-demonstrators

As seen in Table 4, all key variables displayed significant differences based on whether or not respondents participated in 8M demonstrations. Compared with those who did not participate in 8M protests, demonstrators were found to display greater scores in each of the explanatory variables (behavioral and emotional synchrony, positive, and self-transcendent emotions) and the outcomes or indicators of personal and social well-being (contact with values, social cohesion and integration, personal and collective growth, and expectations of participation in the women’s social movement). Sociodemographic variables such as gender, age, and political positioning scale have been controlled for. It is worth noting that the largest differences were observed in the experience of intense emotions (η^2^ = 0.219), pro-women behavior (η^2^ = 0.195), situated social identity (η^2^ = 0.181), and identity fusion with demonstrators (η^2^ = 0.195). The differences between female protesters and non-protesters (followers) are equally significant in all variables when the sample of female participants is analyzed. The female demonstrators display the highest score in all the variables studied (see Supplementary Table 4). The small sample of male and non-binary demonstrators does not allow an effective means comparison.

**TABLE 4:** Differences related to participation and gender in criterion variables.

	Participation-related differences					Gender differences					
	Demonstrators	Non-demonstrators	*F*	*p*	η^2^	Female	Male	Non-binary	*F*	*p*	η^2^
Variables	*M* (*DT*)	*M* (*DT*)				*M* (*DT*)	*M* (*DT*)	*M* (*DT*)			
1. Behavioral synchrony	5.5 (1.24)	4.87 (1.56)	122.989	< 0.001	0.044	5.23 (1.44)^b^	4.76 (1.59)^a^	5.24 (1.37)	17.050	<0.001	0.013
2. Perceived emotional synchrony	5.82 (1.15)	5.39 (1.66)	40.663	< 0.001	0.015	5.66 (1.41)^b^	5.06 (1.75)^a^	5.44 (1.64)	28.357	<0.001	0.021
3. Intense positive emotions	6.03 (1.09)	4.20 (2.01)	693.241	< 0.001	0.207	5.15 (1.83)^b^	3.85 (2.02)^a^	5.53 (1.46)^b^	85.616	<0.001	0.061
4. Self-transcendent emotions	6.18 (0.98)	4.64 (2.02)	529.356	< 0.001	0.166	5.45 (1.72)^b^	4.27 (2.08)^a^	5.66 (1.57)^b^	77.126	<0.001	0.055
5. Self-transcendent experience	5.89 (1.17)	4.62 (1.90)	360.784	< 0.001	0.120	5.30 (1.67)^b^	4.25 (1.93)^a^	5.59 (1.45)^b^	64.828	<0.001	0.047
6. Situated social identity	5.99 (1.13)	4.24 (2.12)	584.689	< 0.001	0.181	5.17 (1.89)^b^	3.80 (2.07)^a^	5.39 (1.69)^b^	89.645	<0.001	0.063
7. Identity fusion demonstrators	4.12 (0.97)	2.94 (1.33)	554.985	< 0.001	0.173	3.57 (1.29)^b^	2.68 (1.26)^a^	3.39 (1.34)^b^	81.544	<0.001	0.058
8. Identity fusion feminist	4.19 (0.94)	3.03 (1.37)	558.975	<0.001	0.174	3.66 (1.30)^b^	2.65 (1.27)^a^	4.03 (1.31)^b^	106.437	<0.001	0.074
9. Solidarity with women	6.55 (0.73)	5.78 (1.47)	230.200	<0.001	0.080	6.18 (1.23)^b^	5.63 (1.43)^a^	6.21 (1.31)^b^	33.810	<0.001	0.025
10. Identity fusion women	4.18 (0.88)	3.92 (1.05)	28.412	<0.001	0.011	4.13 (0.96)^b^	3.50 (0.97)^a^	3.91 (1.18)^b^	72.084	<0.001	0.052
11. Collective efficacy	6.30 (0.89)	5.65 (1.52)	169.220	<0.001	0.060	5.98 (1.27)^b^	5.57 (1.60)^a^	6.13 (1.33)^b^	16.251	<0.001	0.012
12. Positive individual growth	4.53 (1.40)	3.65 (1.72)	218.086	<0.001	0.076	4.14 (1.60)^b^	3.31 (1.75)^a^	4.18 (1.75)^b^	44.520	<0.001	0.032
13. Positive collective growth	5.18 (0.98)	4.24 (1.55)	281.587	<0.001	0.096	4.75 (1.33)^b^	3.93 (1.66)^a^	4.84 (1.38)^b^	60.583	<0.001	0.044
14. Pro-women behavior	4.08 (0.83)	3.00 (1.25)	642.991	<0.001	0.195	3.58 (1.16)^b^	2.68 (1.24)^a^	4.00 (1.10)^b^	103.079	<0.001	0.072

*Different superscripts represent significant differences (at least p < 0.05) conducted as post-hoc dynamic message signs (DMS) tests. n (demonstrators) = 1,091; n [non-demonstrators = 1,568; n (female) = 2,212; n (male) = 414; n (non-Binary) = 33]. In participation-related differences, gender, age, and political positioning scale have been controlled for. In gender differences, age and political positioning scale have been controlled for.*

##### Gender Differences

The gender differences for each key variable score for the total sample are listed in Table 4. In general, when compared with male, both female and non-binary gender respondents were found to score significantly higher on most of the variables of interest (i.e., intense positive emotions, self-transcendent emotions, self-transcendent experience, situated social identity, identity fusion with demonstrators, identity fusion with feminists, solidarity toward women, collective efficacy, positive individual growth, positive collective growth, and pro-woman behavior). Sociodemographic variables such as age and political positioning have been controlled for. The largest differences were found for identity fusion with feminists (η^2^ = 0.072), pro-woman behavior (η^2^ = 0.072), situated social identity (η^2^ = 0.063), and experience of intense positive emotions (η^2^ = 0.062). When compared with male and non-binary gender respondents, female demonstrators were also found to display greater levels of identity fusion with women (η^2^ = 0.042). This was also the case for intense positive emotions (η^2^ = 0.032), self-transcendent emotions (η^2^ = 0.029), and PES (η^2^ = 0.025); overall, the effect size of gender differences was small. Differences between female and male participants were significant (*p* < 0.001) regarding such variables, except collective efficacy and positive individual growth. Please see Supplementary Material for more details (Table 5). These results confirm H3, showing that both demonstrators and women (in comparison with non-demonstrators and men) displayed higher scores in all variables.

**TABLE 5:** Pooled effect size between PES and criterion variables.

	Effect size 95% CI			Heterogeneity			Fail-safe *N*	Egger’s regression		
Variables	*r*	Low	Up	*Q*(8)	*p*	*I*^2^	*n*	*Intercept*	*t*(7)	*p*
1. Behavioral synchrony	0.64	0.586	0.695	40.57	<0.001	80.28	3,266	–1.79	0.99	0.355
2. Intense positive emotions	0.61	0.567	0.657	23.84	0.002	66.44	2,800	0.41	0.28	0.788
3. Self-transcendent emotions	0.65	0.599	0.697	33.58	<0.001	76.18	3,305	–0.20	0.11	0.923
4. Self-transcendent experience	0.65	0.602	0.697	31.83	<0.001	74.87	3,403	–2.25	1.51	0.175
5. Situated social identity	0.59	0.517	0.652	51.35	<0.001	84.42	2,632	–2.81	1.48	0.183
6. Identity fusion demonstrators	0.43	0.365	0.492	28.05	<0.001	71.48	1,184	–1.62	1.09	0.310
7. Identity fusion feminist	0.40	0.312	0.476	45.46	<0.001	82.40	1,010	–1.79	0.93	0.383
8. Solidarity with women	0.43	0.361	0.489	28.31	<0.001	71.74	1,179	–2.11	1.50	0.176
9. Identity fusion women	0.24	0.201	0.286	10.22	0.250	21.72	328	–0.09	0.09	0.927
10. Collective efficacy	0.40	0.368	0.430	3.42	0.905	0.00	932	–0.79	1.66	0.140
11. Positive individual growth	0.41	0.335	0.478	34.94	<0.001	77.10	1,087	–2.52	1.66	0.141
12. Positive collective growth	0.47	0.407	0.523	25.23	0.001	68.30	1,431	–2.03	1.55	0.166
13. Pro-women behavior	0.41	0.333	0.483	38.82	<0.001	79.39	1,099	–2.42	1.47	0.185

*N = 2,843, k = 9, number of studies included in the analysis. Fail-safe N indicates Rosenthal’s fail-safe N analysis. PES, perceived emotional synchrony.*

### Pooled Effect Sizes of Perceived Emotional Synchrony by Countries

The analysis of correlations by country displayed, in general, positive relations between PES and the criterion variables. Psychosocial mechanisms are positively and significantly associated with PES, with correlations between *r* = 0.45 and *r* = 0.77 in all countries included in this research. Descriptive statistics and correlations by country may be viewed in Supplementary Table 2.

We next calculated the pooled effects of *r* of PES with all key variables. The data obtained revealed that PES displayed moderate-to-high positive and significant relationships with all variables (see Table 5).

PES showed the strongest associations with self-transcendent experience (*r* = 0.65) and emotions (*r* = 0.65), behavioral synchrony (*r* = 0.65), and intense positive emotions (*r* = 0.61). Furthermore, less intense but strong relationships were found for PES with situated social identity (*r* = 0.59), positive collective growth (*r* = 0.47), identity fusion with demonstrators (*r* = 0.43), social identity dimension of solidarity toward women (*r* = 0.43), pro-woman behavior (*r* = 0.41), positive individual growth (*r* = 0.41), collective efficacy (*r* = 0.41), and identification or identity fusion with feminists (*r* = .40). The lowest of the effects was found in relation to identity fusion with women (*r* = 0.27). All pooled effect sizes were statistically significant^3^.

The analysis of heterogeneity reveals the existence of two sizes of homogeneous effect in the nine countries of analysis. The first and with less variability is in the relation between PES and group efficiency [*r* = 0.41; *Q*(8) = 4.23, *p* = 0.181; *I*^2^ = 0.00], and the other is regarding the relationship between identity fusion and/or proximity to women [*r* = 0.25; *Q*(8) = 4.23, *p* = 0.836; *I*^2^ = 29.67]. It is important to note that the analysis yielded non-significant Egger’s regressions in all cases (see Table 5), which excludes the existence of asymmetrical relations between effect sizes and standard errors. This observation, along with solid Rosenthal’s fail-safe *N* tests values (ranging from 338 in PES-identity fusion with women to 8,907 in PES-behavioral synchrony) suggests consistent effects of the associations as well as the absence of potential selection biases with the samples used.

### Model of Multiple Serial Mediation

We applied a model of multiple serial mediation (Model 6; Hayes’ PROCESS Macro for SPSS; Hayes, 2013). However, as expected, there were more participants in the female category than in the male or other categories. To control this circumstance, multiple serial mediation was carried out using only women. The total effect of participation in 8M demonstrators (vs. non-demonstrators) on each dependent variable and total indirect effects are provided in Table 6. The demonstrators (vs. non-demonstrators) in the 8M protests were significantly related to higher scores on all dependent variables. These effects ranged from *b* = 0.19 on identity fusion with women to *b* = 0.93 on pro-woman behavior. Furthermore, participation (vs. non-participation) in 8M demonstrations was also significantly associated with all proposed mediating variables [with the exception of PES (*b* = −0.04)]. In particular, as seen in Figure 1, demonstrators (vs. non-demonstrators) at the protests were related to higher behavioral synchrony (*b* = 0.52), intense positive emotions (*b* = 0.80), and self-transcendent emotions (*b* = 0.07). Regarding the connection between the mediating and dependent variables, our results revealed that not all paths emerged as significant (see Table 6). Our results showed that behavioral synchrony was only significantly related to increased solidarity toward women (*b* = 0.06) and self-transcendent experience (*b* = 0.04). PES was associated with a greater self-transcendent experience (*b* = 0.22), situated social identity (*b* = 0.14) and identity fusion with demonstrators (*b* = 0.06), and feminists (*b* = 0.05). Similar results were found in the case of intense emotions. The experience of intense emotions was related to higher scores on the same variables (with coefficients ranging from 0.12 in self-transcendence experience to 0.31 in identification with feminists), as well as with increased pro-woman behavior (*b* = 0.48). Unlike the precedent mediators, self-transcendent emotion levels were significantly associated with all dependent variables (standardized coefficients ranged from 0.24 in identity fusion with feminists to 0.53 in self-transcendent experience and collective efficacy).

**TABLE 6:** Sequential mediation; total indirect effect and total effect.

DV	e	f	j	k	l	o	p	Total indirect effect	Total effect
Self-transcendent experience	0.53**	0.05*	0.04**	0.22**	0.12**	0.02	−0.03*	0.70 (0.03) 95% CI [0.63,0.76]	0.76 (0.06) 95% CI [1.13, 1.40]
Situated social identity	0.48**	0.17**	0.01	0.14**	0.24**	−0.02*	0.03**	0.73 (0.03) 95% CI [0.67,0.80]	0.91 (0.07) 95% CI [1.59, 1.89]
IF demonstrators	0.32**	0.35**	0.002	0.06*	0.25**	–0.006	–0.009	0.57 (0.02) 95% CI [0.51,0.63]	0.92 (0.05) 95% CI [1.10, 1.30]
Identity fusion feminist	0.24**	0.34**	–0.02	0.05*	0.31**	–0.02	−0.04*	0.54 (0.02) 95% CI [0.48,0.60]	0.88 (0.05) 95% CI [1.04, 1.25]
Solidarity with women	0.51**	0.06	0.06*	0.03	0.05	–0.006	0.03*	0.56 (0.03) 95% CI [0.50,0.63]	0.63 (0.05) 95% CI [0.67, 0.88]
Identity fusion women	0.24**	−0.11*	0.01	0.05	0.06	0.02	0.0003	0.30 (0.03) 95% CI [0.24,0.36]	0.19 (0.04) 95% CI [0.10, 0.27]
Collective efficacy	0.52**	0.01	0.03	0.03	0.03	–0.009	–0.01	0.53 (0.03) 95% CI [0.47,0.60]	0.55 (0.05) 95% CI [0.60, 0.82]
Individual growth	0.40**	0.34**	–0.01	0.03	0.21**	−0.07**	−0.04*	0.58 (0.02) 95% CI [0.52,0.64]	0.93 (0.04) 95% CI [0.99, 1.17]
Collective growth	0.53**	–0.01	–0.02	0.05*	0.08*	–0.01	−0.08**	0.57 (0.03) 95% CI [0.50,0.63]	0.55 (0.06) 95% CI [0.76, 1.02]
Pro-women behavior	0.48**	0.11*	−0.03*	0.11**	0.12**	–0.01	−0.05**	0.58 (0.03) 95% CI [0.52,0.65]	0.69 (0.05) 95% CI [0.82, 1.04]

95% CI. Standardized effects (partially standardized total indirect effect). f = total direct effect. N = 2,217. *p ≤ 0.05; **p ≤ 0.001. DV, dependent variable.

All total indirect effects emerged as significant because the 0 value was not included in any of the CIs generated. Therefore, our results confirmed that behavioral synchrony, PES, intense positive emotions, and self-transcendent emotions successively mediate the associations of participation (vs. non-participation) in 8M demonstrations with all dependent variables. Indirect effects ranged from *b* = 0.30 (in the case of identity fusion with women) to *b* = 0.73 (in situated social identity). Overall, participation in 8M protests was indirectly related to the self-transcendence experience, situated social identity, identity fusion with demonstrators, feminists and women, solidarity toward women, collective efficacy, and pro-woman behavior via its linkages with behavioral synchrony, PES, intense positive emotions, and self-transcendent emotions. After the effects of the mediator variables were controlled for, the direct effects of participation (vs. non-participation) on solidarity toward women and collective efficacy were not significant, thus indicating the existence of complete mediations. Partial meditations were found for the rest of dependent variables (i.e., self-transcendent experience, situated social identity, identity fusion with demonstrators, feminists, and women, and pro-woman behavior). Hypothesis 4 has only been partially confirmed.

## Conclusion

Globally, this study is consistent with Durkheim’s theoretical proposal (Durkheim, 1912/2008), later developed by Collins (2004), analyzing the 8M demonstrations from the perspective of collective rituals. Participation in these ritualized collective actions is related to a series of positive effects on well-being, both individually and collectively. At the same time, such the participation in these ritualized collective actions is linked to a series of psychosocial mechanisms (behavioral and attentional synchrony, PES, and intense self-referential and self-transcendent emotions), which have been empirically studied in previous research with other collective rituals and meetings (e.g., Páez et al., 2015; Gabriel et al., 2017, 2020; Wlodarczyk et al., 2020).

The results obtained reveal that participation in collective rituals and gatherings, with emotional sharing and convergence, reinforces most of the attributes of subjective and psychological well-being (Vázquez and Hervás, 2009; Diener et al., 2011; Ryff, 2014). Compared with non-demonstrators (followers), demonstrators report higher levels of well-being, such as subjective well-being or personal affective well-being (positive and self-transcendent emotions), a greater meaning in life or sharing transcendental values (agreement and contact with values), a sense of contextual and social identity that is coherent and strong (social identification and fusion identity), mastery or high collective self-efficacy or positive relations with others, and social integration by means of participating in a women’s movement. Some sociodemographic variables such as gender, age, and political positioning were controlled for in an effort to avoid the effect of previous differences between the comparison groups. In the comparison by gender, there were higher female scores, especially regarding antecedents and PES (albeit the effect size is small). This partially confirms that participation in 8M demonstrations had a greater effect on women (female). It is likely that the experience is intensified when one recognizes herself as part of the target collective of the event.

All the explanatory variables, particularly attentional and behavioral synchrony, PES, and positive emotions (self-referential and self-transcendent), are related to personal and social well-being, social integration, and empowerment. In addition, the PES was significantly but heterogeneously associated with the vast majority of the criterion variables and predicted them, except identity fusion with women and collective efficacy, which revealed homogeneous effect sizes in all countries. A recent meta-analysis on collective effervescence (Rimé et al., unpublished) supports the fact that there is a stable and solid association of PES and results related to personal and social well-being, agreement with values, social integration, empowerment, and prosocial behavior. In line with Wlodarczyk et al.’s (2020, in this monograph) work, behavioral and attentional synchrony, PES antecedents, appeared to be related with a large effect size. In the same fashion, in line with previous related research, the results revealed that PES is highly associated with the intense positive emotional experience experienced during collective participation in all samples, the joy created when sharing with others (Páez et al., 2015; Wlodarczyk et al., 2020; Zabala et al., 2020), and with self-transcendent emotions (Cusi et al., 2018; Fiske et al., 2019; Pizarro et al., 2018). All psychosocial mechanisms studied showed large effect sizes (>0.60). Moreover, PES has a positive, large-magnitude relation with the experience of transcendence, generated upon contact with collective symbols and values (van Cappellen and Rimé, 2014; Gabriel et al., 2020). Large effect sizes were found, although more moderate in general, with variables related to cohesion and social integration, such as social identity or identity fusion, in concordance with previous research (e.g., Khan et al., 2015; Bäck et al., 2018). In general, these were greater in magnitude when in relation to the variables of situated or contextual social identity, meaning identification with other participants. On the other hand, in a broader sense of social identity (Gasparre et al., 2010), meaning solidarity toward women, there was a large effect size, but there was no identity fusion with women, which revealed a medium-sized effect, although an effect that was homogeneous in cross-cultural terms. With the variables related to empowerment, PES displayed positive and significant relations with effect sizes above 0.40, showing that participation promotes the perception of collective efficacy in a homogeneous fashion at a transnational level, as well as beliefs of both individual and collective growth (Páez et al., 2015; Wlodarczyk et al., 2020) in a heterogeneous fashion. This is also associated with a large, heterogeneous magnitude with the intention to help women.

In this regard, the analysis of sequential mediation conducted on the sample of women supports a model wherein participation facilitates attentional and behavioral synchrony, sparks collective effervescence or PES, boosts positive and transcendent emotions, facilitates agreement and contact with values and the sacred, and drives all the results. Specifically, these results indicate that participation in demonstrations reinforces positive, self-transcendental emotions above emotional synchrony. Participation in demonstrations through PES and intense positive emotions feeds into contact with values, situated and in-group social identity, and identity fusion. Lastly, participation through feelings of self-transcendence reinforces all the results. The results underscore the importance of experiences and emotions of self-transcendence because of the PES, which encourages the positive effects of collective meetings. Recent research provides empirical evidence in this same direction (Cusi et al., 2018; Pizarro et al., 2018). However, some results are striking, for example, the suppression effect found in identity fusion with women, which may be due to the characteristics intrinsic to the ritualized 8M demonstration, where one of the march’s main pillars are women’s claims and the active fight for civil rights.

We acknowledge the main limitations of this study First, we worked with a convenience sample, which is a limitation regarding inferences about the general population. Moreover, data collection in natural contexts makes more difficult to obtain large samples, diverse samples (age and gender), balanced numbers between countries (Brazil, Portugal, and Ecuador have smaller sample sizes), and types of participants in each country (Colombia and Brazil have lower percentages of demonstrators). Given the nature of 8M, the female population is over-represented, while male demonstrators are very few. Second, due to the correlational nature of the study and the characteristics mentioned above, some of the results may reflect previous differences between demonstrators and non-demonstrators, not necessarily linked to participation in the demonstrations, even with the statistical control that we undertook (age, gender, and political positioning). We suggest that future research should include pre–post measures or control groups to minimize this limitation. Third, the sample also appears to be biased in favor of those willing to participate in in 8M 2020 (demonstrations and non-demonstrations) and the study, as well as toward a representative profile, given that attention was focused on assessing the impact of participation. It would be useful to include other study groups (e.g., control group) to assess these effects, beyond the subjective perception of participation, and to include the impact of 8M on the community in general. This aspect, in addition to a longitudinal design, would allow control for possible prior differences between the compared groups (e.g., demonstrations vs. non-demonstrations); in this correlational study, the effects of sociodemographic variables and political orientation have been controlled for. Lastly, collective effervescence and its relationship with the mid- and long-term effects of participation would be one of the objectives to include in future research. According to previous literature, these effects are limited over time. Durkheim (1912/2008) and Páez et al. (2015) indicated that a necessary condition for a collective ritual’s effects to persist over time is regularity (frequency).

Despite the limitations of this study, we believe that significant contributions derive from the current research study. First, we are not aware of previous studies that analyze quantitative relationships between psycho-emotional effects of participating in international social mobilizations, as the ritualized demonstrations of 8M. There is a scar of peer-reviewed quantitative studies on the 8M participation and its psycho-emotional correlates, or its relation to variables such as individual and collective well-being, social cohesion, or individual and collective growth, among others. Second, this work shows the relevance of psycho-emotional mechanisms in both participants and followers. This aspect has been largely neglected in the scientific literature (Hobson et al., 2018), given that being an audience through mass media and social networks (followers) is a new form of participation. Indeed, it is a step forward in the long and active line of research on the participation in rituals and collective meetings and collective effervescence study, especially, being the first time that it is included in a natural context an integrative measurement proposal made by Wlodarczyk et al. (2020). From a social perspective, we believe that it is relevant to emphasize the positive aspects (well-being and collective well-being, social integration, collective empowerment, and behavioral intention to support others) associated with participation in ritualized demonstrations.

In sum, this research provides valuable insight to understand the psychological and emotional mechanisms (and their relationships) generated during collective participation in ritualized collective actions such as 8M demonstrations. These findings could also shed light on the relevant role of the experience of collective effervescence that improves personal and social well-being, social cohesion and integration, and empowerment of all participants, with more intensity in the reference group (in this case, women). Finally, the shared cognitive and emotional experience in ritualized collective actions serves to renew commitment to the community, to improve well-being, and to strengthen both the individuals and the groups involved. These shared emotional experiences may prove to be useful tools to promote social change and the transformation of societies, with the ultimate goal of working toward equality and prosocial models through collective political participation.

## Data Availability Statement

Further inquiries can be directed to the corresponding authors. The raw data supporting the conclusions of this article will be made available by the authors, without undue reservation.

## Ethics Statement

All study participants read and accepted informed consent. The data recorded was alphanumerically code to ensure anonymity following the Organic Law on the Protection of Personal Data (BOE-A-2018-16673), and compliance with the regulation of the Ethics Committee for Research Involving Human Beings (CEISH) by the University of the Basque Country.

## Author Contributions

LZ, PC-A, LM, JP, AW, NB, SC, SP, GN-C, IA-A, and BT-G planned and contributed to this cross-cultural study, performed the questionnaires, drafted the manuscript, performed the calculations, discussed the results, and commented and revised on the manuscript. All authors coordinated the sample collection in their residence areas/countries, and contributed to the discussion of the results.

## Conflict of Interest

The authors declare that the research was conducted in the absence of any commercial or financial relationships that could be construed as a potential conflict of interest.
