# Supplementary material for: Collective Effervescence, Self-Transcendence, and Gender Differences in Social Well-Being During 8 March Demonstrations
Source: Front Psychol. 2020 Dec 11;11:607538. doi: 10.3389/fpsyg.2020.607538 (PMC7759529; doi:10.3389/fpsyg.2020.607538)
Supplement: Supplementary file 1 [file Table_1.DOCX]

# Supplementary Material

**Table I.**

*Socio-demographics data by country*

|  |  | **Age** | | **Gender** | | | **Education^a^** | | **Political positioning^b^** | | | |
| --- | --- | --- | --- | --- | --- | --- | --- | --- | --- | --- | --- | --- |
| **Country** | ***N*** | ***M*** | ***SD*** | **F** | **M** | **Non-Binary** | **High** | **Low** | **Left** | **Middle** | **Right** | **No positioning** |
| **SPAIN** | 461 | 36.87 | (14.17) | 84.0% | 14.7% | 1.3% | 85.6% | 14.4% | 65.8% | 23.3% | 1.4% | 9.5% |
| Demonstrators | 238 | 36.84 | (14.89) | 92.4% | 5.9% | 1.7% | 88.7% | 11.3% | 85.6% | 13.9% | 0.5% | 0.0% |
| Non-demonstrators | 223 | 36.90 | (13.40) | 75.3% | 23.8% | 0.9% | 82.0% | 18% | 47.3% | 32.0% | 2.3% | 18.5% |
| **MEXICO** | 1047 | 29.98 | (9.39) | 86.0% | 13.6% | 0.4% | 97.2% | 2.8% | 20.8% | 38.5% | 2.2% | 38.5% |
| Demonstrators | 413 | 31.16 | (9.01) | 96.4% | 2.9% | 0.7% | 97.4% | 2.6% | 35.2% | 61.6% | 3.1% | 0.0% |
| Non-demonstrators | 634 | 29.21 | (9.56) | 79.2% | 20.5% | 0.3% | 96.0% | 4.0% | 12.7% | 25.5% | 1.7% | 60.1% |
| **PERU** | 248 | 29.64 | (11.94) | 75.3% | 21.2% | 3.3% | 95.0% | 5.0% | 28.4% | 41.2% | 7.4% | 23.0% |
| Demonstrators | 81 | 30.63 | (13.73) | 91.4% | 3.7% | 4.9% | 94.9% | 5.1 % | 61.8% | 36.8% | 1.3% | 0.0% |
| Non-demonstrators | 167 | 29.16 | (10.98) | 67.7% | 29.9% | 2.4% | 94.6% | 5.4% | 13.2% | 43.1% | 10.2% | 33.5% |
| **COLOMBIA** | 203 | 23.19 | (9.84) | 75.3% | 23.2% | 1.6% | 75.9% | 24.1% | 9.5% | 32.8% | 3.0% | 54.7% |
| Demonstrators | 23 | 24.04 | (6.83) | 78.3% | 17.4% | 4.3% | 85.7% | 14.3% | 61.9% | 38.1% | 0.0% | 0.0% |
| Non-demonstrators | 180 | 23.08 | (10.17) | 76.1% | 22.8% | 1.1% | 72.1% | 27.9% | 3.3% | 32.2% | 3.3% | 61.2% |
| **BRAZIL** | 72 | 35.56 | (14.75) | 88.9% | 9.7% | 1.4% | 87.5% | 7.0% | 75.4% | 11.6% | 0.0% | 13.0% |
| Demonstrators | 17 | 42.41 | (15.14) | 100% | 0.0% | 0.0% | 0.0% | 100% | 92.9% | 7.1% | 0.0% | 0.0% |
| Non-demonstrators | 55 | 33.44 | (14.10) | 85.5% | 12.7% | 1.8% | 0.0% | 100% | 70.9% | 12.7% | 0.0% | 16.4% |
| **PORTUGAL** | 67 | 33.42 | (13.86) | 77.6% | 11.9% | 10.4% | 0.0% | 85.0% | 73.2% | 8.9% | 3.6% | 14.3% |
| Demonstrators | 42 | 34.33 | (13.52) | 76.2% | 11.9% | 11.9% | 0.0% | 100% | 87.1% | 3.2% | 0.0% | 9.7% |
| Non-demonstrators | 25 | 31.88 | (14.55) | 80.0% | 12.0% | 8.0% | 0.0% | 100% | 56.0% | 16.0% | 8.0% | 20.0% |
| **ARGENTINA** | 209 | 22.04 | (5.32) | 87.0.0% | 12.6% | 0.5% | 75.2% | 24.8% | 4.9% | 48.8% | 19.7% | 26.6% |
| Demonstrators | 50 | 23.56 | (6.57) | 94.0% | 4.0% | 2.0% | 76.1% | 24.9% | 13.6% | 81.8% | 4.6% | 0.0% |
| Non-demonstrators | 159 | 21..56 | (4.78) | 84.9% | 15.1% | 0.0% | 74.1% | 25.9% | 2.5% | 39.6% | 23.9% | 34.0.0% |
| **CHILE** | 478 | 29.61 | (11.31) | 86.5% | 12.4% | 1.1% | 89.9% | 10.1% | 40.9% | 45.9% | 2.6% | 10.6% |
| Demonstrators | 358 | 29.87 | (10.92) | 96.9% | 2.2% | 0.8% | 90.6% | 9.4% | 49.7% | 49.3% | 1.0% | 0.0% |
| Non-demonstrators | 120 | 28.82 | (12.41) | 55.0% | 43.3% | 1.7% | 86.7% | 13.3% | 19.2% | 37.5% | 6.7% | 36.7% |
| **ECUADOR** | 103 | 34.65 | (10.22) | 78.6% | 19.4% | 1.9% | 95.1% | 4.9% | 47.0% | 36.0% | 2.0% | 15% |
| Demonstrators | 58 | 36.41 | (11.43) | 89.7% | 6.9% | 3.4% | 94.7% | 5.3% | 57.1% | 41.1% | 1.8% | 0.0% |
| Non-demonstrators | 45 | 32.38 | (7.99) | 64.4% | 35.6% | 0.0% | 95.6% | 4.4% | 34.1% | 29.5% | 2.3% | 34.1% |
| *Note*: Valid % are reported. ^a^Dicotomized from four levels (High = *University*. Low = *Primary. Secondary. Tertiary*). ^b^Continuous scale 1 - 7 categorized (*Left* = 1 and 2. *Middle* = 3. 4 and 5. *Right* = 6 and 7). | | | | | | | | | | | | |
