# Supplementary material for: Collective Effervescence, Self-Transcendence, and Gender Differences in Social Well-Being During 8 March Demonstrations
Source: Front Psychol. 2020 Dec 11;11:607538. doi: 10.3389/fpsyg.2020.607538 (PMC7759529; doi:10.3389/fpsyg.2020.607538)
Supplement: Supplementary file 2 [file Table_2.DOCX]

**Table II**

*Confirmatory Factor Analysis fits for each scale.*

| ***Instrument*** | ***Items*** | ***ꭓ^2^*** | **gl** | **CFI** | **RMSEA** | **IC 90% RMSEA** | **SRMR** | **Ω** |
| --- | --- | --- | --- | --- | --- | --- | --- | --- |
| Perceived Emotional Synchrony | 6 | 35.580 | 8 | .995 | .052 | .035, .070 | .011 | .818 |
| Intense Positive Emotions | 3 | 0.001 | 0 | 1.00 | .000 | .000, .000 | .001 | .933 |
| Self-transcendent Emotions | 5 | 23.223 | 4 | .999 | .041 | .026, .058 | .004 | .956 |
| Self-transcendent Experience | 4 | 0.165 | 1 | 1.00 | .000 | .000, .037 | .001 | .927 |
| Situated Social Iden·tity | 3 | 0.001 | 0 | 1.00 | .000 | .000, .000 | .001 | .946 |
| Solidarity with Women | 3 | 0.001 | 0 | 1.00 | .000 | .000, .000 | .001 | .911 |
| Collective Efficacy | 4 | 2.031 | 1 | 1.00 | .019 | .000, .059 | .003 | .922 |
| Positive Individual Growth | 3 | 0.001 | 0 | 1.00 | .000 | .000, .000 | .001 | .930 |
| Positive Collective Growth | 3 | 0.001 | 0 | 1.00 | .000 | .000, .000 | .001 | .930 |
| Pro-women behavior | 5 | 93.932 | 8 | .994 | .081 | .066, .098 | .017 | .930 |

*Note*. The fit indices utilized were Chi squared (*ꭓ^2^*); Degrees of freedom (*gl*); Comparative Fit Index (CFI);RMSEA, Root Mean Square Error of Approximation (RMSEA); Standardized Root Mean Square Residual (SRMR); y Ω (McDonald’s omega). Seven variables have excluded from this analysis for not having the recommended number of items (>3) to perform the CFA (Participation, Behavioral synchrony[2 items, *r* = .57], Identity Fusion demonstration, Identity Fusion Feminist, Identity Fusion Women, Political Orientation, and Subjective SES)
