# Supplementary material for: Collective Effervescence, Self-Transcendence, and Gender Differences in Social Well-Being During 8 March Demonstrations
Source: Front Psychol. 2020 Dec 11;11:607538. doi: 10.3389/fpsyg.2020.607538 (PMC7759529; doi:10.3389/fpsyg.2020.607538)
Supplement: Supplementary file 3 [file Table_3.DOCX]

**Table III.**

*Correlational analysis with PES and Cronbach alpha for each criterion variable by country*

|  | **Countries (*r*)** | | | | | | | | | |
| --- | --- | --- | --- | --- | --- | --- | --- | --- | --- | --- |
| **VARIABLES** | **Argentina** | **Brazil** | **Chile** | **Colombia** | **Ecuador** | **Spain** | | **Mexico** | **Peru** | **Portugal** |
| Behavioral synchrony | .59** | .61** | .54** | .74** | .56** | .65** | | .71** | .71** | .57** |
| Intense Positive Emotions | .47** | .70** | .58** | .58** | .54** | .62** | | .63** | .69** | .76** |
| Transcendent Emotions | .47** | .77** | .61** | .65** | .54** | .67** | | .67** | .73** | .72** |
| Transcendent experience | .60** | .63** | .62** | .62** | .59** | .65** | | .71** | .77** | .54** |
| Situated social identity | .48** | .62** | .53** | .50** | .46** | .63** | | .68** | .73** | .57** |
| Identity Fusion demonstration | .33** | .32** | .41** | .34** | .46** | .42** | | .49** | .62** | .33** |
| Identity Fusion Feminist | .42** | .32** | .32** | .30** | .47** | .37** | | .49** | .62** | .01 |
| Solidarity with Women | .28** | .19 | .32** | .13 | .34** | .18** | | .24** | .29** | .20 |
| Identity Fusion Women | .42** | .40** | .32 | .37** | .41** | .42** | | .54** | .49** | .40** |
| Collective Efficacy | .41** | .32** | .380* | .37** | .32** | .39** | | .42** | .44** | .42** |
| Positive Individual Growth | .40** | .15 | .45** | .36** | .29** | .35** | | .50** | .60** | .37** |
| Positive Collective Growth | .41** | .39** | .40** | .48** | .42** | .44** | | .55** | .59** | .37** |
| Pro-women behavior | .42** | .15 | .33** | .38** | .35** | .40** | | .52** | .58** | .41** |
| **VARIABLES** | **Scale Alphas (α)** | | | | | | | | | |
| Behavioral synchrony* | .66 (.49) | .56 (.40) | .52 (.36) | .79 (.65) | .71 (.56) | .73 (.58) | | .72 (.57) | .74 (.60) | .68 (.52) |
| Intense Positive Emotions | .92 | .90 | .90 | .93 | .89 | .93 | | .92 | .96 | .91 |
| PES | .81 | .74 | .83 | .84 | .86 | .85 | | .90 | .90 | .85 |
| Transcendent Emotions | .96 | .87 | .92 | .94 | .92 | .94 | | .96 | .97 | .90 |
| Transcendent experience | .88 | .88 | .85 | .93 | .91 | .90 | | .93 | .95 | .91 |
| Situated social identity | .95 | .92 | .91 | .93 | .89 | .93 | | .95 | .96 | .93 |
| Identity Fusion demonstration’s | 1 | 1 | 1 | 1 | 1 | 1 | | 1 | 1 | 1 |
| Identity Fusion Feminist | 1 | 1 | 1 | 1 | 1 | 1 | | 1 | 1 | 1 |
| Solidarity with Women | .91 | .89 | .86 | .90 | .86 | .91 | | .92 | .88 | .80 |
| Identity Fusion Women | 1 | 1 | 1 | 1 | 1 | 1 | | 1 | 1 | 1 |
| Collective Efficacy | .90 | .88 | .90 | .90 | .87 | .91 | | .92 | .93 | .86 |
| Positive Individual Growth | .93 | .90 | .92 | .93 | .91 | .92 | | .92 | .94 | .95 |
| Positive Collective Growth | .89 | .93 | .87 | .92 | .88 | .94 | | .90 | .94 | .94 |
| Pro-women behavior | .89 | .85 | .84 | .89 | .84 | .86 | | .89 | .94 | .85 |
| *N* | 207 | 72 | 475 | 190 | 103 | 457 | | 1032 | 245 | 67 |
| *Note*: Behavioral synchrony*: inside the parentheses reported total correlation of items corrected (2 items). | | | | | | |  |  |  |  |
