# Supplementary material for: Collective Effervescence, Self-Transcendence, and Gender Differences in Social Well-Being During 8 March Demonstrations
Source: Front Psychol. 2020 Dec 11;11:607538. doi: 10.3389/fpsyg.2020.607538 (PMC7759529; doi:10.3389/fpsyg.2020.607538)
Supplement: Supplementary file 4 [file Table_4.DOCX]

**DATA FOR EACH GENDER**

**Table IV.**

*Mean comparison between demonstrators and non-demonstrators for each gender (GML)*

|  |  | **Female** | | | | |  | **Male** | | | | |  | **Non-Binary** | | | | |
| --- | --- | --- | --- | --- | --- | --- | --- | --- | --- | --- | --- | --- | --- | --- | --- | --- | --- | --- |
| ***Variables*** |  | ***M*** | ***SD*** | ***F*** | ***p*** | **η^2^** |  | ***M*** | ***SD*** | ***F*** | ***p*** | **η^2^** |  | ***M*** | ***SD*** | ***F*** | ***p*** | **η^2^** |
| Behavioral synchrony | D's | 5.61 | 1.25 | 156.40 | <.001 | .061 |  | 4.85 | 1.50 | 0.17 | .676 | .000 |  | 5.15 | 1.36 | 0.11 | .742 | .003 |
|  | Non D's | 4.90 | 1.54 |  |  |  |  | 4.75 | 1.61 |  |  |  |  | 4.97 | 2.01 |  |  |  |
|  |  |  |  |  |  |  |  |  |  |  |  |  |  |  |  |  |  |  |
| PES | D's | 5.86 | 1.16 | 41.07 | <.001 | .017 |  | 5.03 | 1.42 | 0.00 | .968 | .000 |  | 5.11 | 1.56 | 0.52 | .476 | .014 |
|  | Non D's | 5.50 | 1.60 |  |  |  |  | 5.04 | 1.79 |  |  |  |  | 5.55 | 2.25 |  |  |  |
|  |  |  |  |  |  |  |  |  |  |  |  |  |  |  |  |  |  |  |
| Intense Positive Emotions | D's | 6.05 | 1.10 | 666.69 | <.001 | .216 |  | 5.06 | 1.54 | 22.04 | <.001 | .049 |  | 5.54 | 1.62 | 0.54 | .465 | .014 |
|  | Non D's | 4.37 | 1.98 |  |  |  |  | 3.69 | 2.02 |  |  |  |  | 5.11 | 2.00 |  |  |  |
|  |  |  |  |  |  |  |  |  |  |  |  |  |  |  |  |  |  |  |
| Transcendent Emotions | D's | 6.21 | 0.99 | 496.37 | <.001 | .170 |  | 5.48 | 1.31 | 2.67 | <.001 | .046 |  | 5.45 | 1.63 | 0.10 | .751 | .003 |
|  | Non D's | 4.81 | 1.96 |  |  |  |  | 4.11 | 2.11 |  |  |  |  | 5.25 | 2.20 |  |  |  |
|  |  |  |  |  |  |  |  |  |  |  |  |  |  |  |  |  |  |  |
| Transcendent experience | D's | 5.92 | 1.20 | 332.54 | <.001 | .121 |  | 5.25 | 1.33 | 15.83 | <.001 | .036 |  | 5.50 | 1.43 | 0.44 | .513 | .012 |
|  | Non D's | 4.76 | 1.86 |  |  |  |  | 4.13 | 1.95 |  |  |  |  | 5.12 | 2.21 |  |  |  |
|  |  |  |  |  |  |  |  |  |  |  |  |  |  |  |  |  |  |  |
| Situated social identity | D's | 6.01 | 1.16 | 536.15 | <.001 | .181 |  | 5.26 | 1.41 | 3.38 | <.001 | .067 |  | 5.43 | 1.53 | 1.02 | .32 | .017 |
|  | Non D's | 4.42 | 2.10 |  |  |  |  | 3.62 | 2.08 |  |  |  |  | 4.80 | 2.39 |  |  |  |
|  |  |  |  |  |  |  |  |  |  |  |  |  |  |  |  |  |  |  |
| Identity Fusion demonstration’s | D's | 4.17 | 0.95 | 571.47 | <.001 | .192 |  | 3.24 | 1.14 | 1.98 | .001 | .025 |  | 3.50 | 1.29 | 1.22 | .277 | .032 |
|  | Non D's | 3.04 | 1.34 |  |  |  |  | 2.61 | 1.27 |  |  |  |  | 3.00 | 1.51 |  |  |  |
|  |  |  |  |  |  |  |  |  |  |  |  |  |  |  |  |  |  |  |
| Identity Fusion Feminist | D's | 4.21 | 0.95 | 478.43 | <.001 | .166 |  | 3.55 | 1.05 | 29.99 | <.001 | .066 |  | 4.00 | 1.32 | 0.32 | .573 | .009 |
|  | Non D's | 3.15 | 1.38 |  |  |  |  | 2.54 | 1.26 |  |  |  |  | 3.73 | 1.58 |  |  |  |
|  |  |  |  |  |  |  |  |  |  |  |  |  |  |  |  |  |  |  |
| Solidarity with Women | D's | 6.57 | 0.76 | 214.46 | <.001 | .084 |  | 6.25 | 0.91 | 1.19 | .002 | .044 |  | 6.35 | 1.00 | 1.67 | .205 | .024 |
|  | Non D's | 5.85 | 1.46 |  |  |  |  | 5.56 | 1.47 |  |  |  |  | 5.71 | 2.02 |  |  |  |
|  |  |  |  |  |  |  |  |  |  |  |  |  |  |  |  |  |  |  |
| Identity Fusion Women | D's | 4.20 | 0.87 | 16.68 | <.001 | .007 |  | 3.55 | 0.97 | 0.14 | .709 | .000 |  | 3.67 | 1.24 | 0.42 | .523 | .011 |
|  | Non D's | 4.04 | 1.05 |  |  |  |  | 3.49 | 0.98 |  |  |  |  | 3.93 | 1.28 |  |  |  |
|  |  |  |  |  |  |  |  |  |  |  |  |  |  |  |  |  |  |  |
| Collective Efficacy | D's | 6.32 | 0.91 | 148.79 | <.001 | .060 |  | 6.06 | 0.95 | 4.90 | .027 | .011 |  | 6.16 | 1.12 | 0.52 | .475 | .014 |
|  | Non D's | 5.69 | 1.48 |  |  |  |  | 5.52 | 1.66 |  |  |  |  | 5.80 | 1.98 |  |  |  |
|  |  |  |  |  |  |  |  |  |  |  |  |  |  |  |  |  |  |  |
| Positive Individual Growth | D's | 4.57 | 1.38 | 143.86 | <.001 | .058 |  | 4.12 | 1.55 | 11.55 | .001 | .027 |  | 4.42 | 1.56 | 1.83 | .184 | .048 |
|  | Non D's | 3.80 | 1.68 |  |  |  |  | 3.22 | 1.75 |  |  |  |  | 3.62 | 2.07 |  |  |  |
|  |  |  |  |  |  |  |  |  |  |  |  |  |  |  |  |  |  |  |
| Positive Collective Growth | D's | 5.21 | 0.98 | 254.79 | <.001 | .099 |  | 4.72 | 1.22 | 12.47 | <.001 | .029 |  | 5.14 | 0.63 | 4.19 | .048 | .141 |
|  | Non D's | 4.37 | 1.48 |  |  |  |  | 3.84 | 1.68 |  |  |  |  | 4.20 | 2.08 |  |  |  |
|  |  |  |  |  |  |  |  |  |  |  |  |  |  |  |  |  |  |  |
| Pro-women behavior | D's | 4.09 | 0.84 | 481.85 | <.001 | .172 |  | 3.72 | 0.85 | 4.30 | <.001 | .087 |  | 4.27 | 0.80 | 5.27 | .028 | .128 |
|  | Non D's | 3.13 | 1.22 |  |  |  |  | 2.55 | 1.24 |  |  |  |  | 3.43 | 1.46 |  |  |  |

*Note:* D's = Demonstrators; Non D's = Non-Demonstrators, followers. Female: *n*(Demonstrators) = 1205. *n*(Non-Demonstrators) = 1222 / Male: D's = 48. Non D's =376 / Non-binary: D's = 24. Non D's =15.
