# Supplementary material for: Collective Effervescence, Self-Transcendence, and Gender Differences in Social Well-Being During 8 March Demonstrations
Source: Front Psychol. 2020 Dec 11;11:607538. doi: 10.3389/fpsyg.2020.607538 (PMC7759529; doi:10.3389/fpsyg.2020.607538)
Supplement: Supplementary file 5 [file Table_5.DOCX]

m

**Table V.**

*Gender comparison by participation type (GML).*

|  | **Non-Demonstrators** | | | | | |  | **Demonstrators** | | | | | |
| --- | --- | --- | --- | --- | --- | --- | --- | --- | --- | --- | --- | --- | --- |
|  | **Female** | **Male** | **Non-Binary** |  |  |  |  | **Female** | **Male** | **Non-Binary** |  |  |  |
|  | ***M (SD)*** | ***M (SD)*** | ***M (SD)*** | ***F*** | ***p*** | **η2** |  | ***M (SD)*** | ***M (SD)*** | ***M (SD)*** | ***F*** | ***p*** | **η2** |
| 1. Behavioral synchrony | 4.90 (1.54) | 4.75 (1.61) | 4.97 (2.01) | 1.37 | .255 | .002 |  | 5.61 (1.25)b | 4.85 (1.50)a | 5.15 (1.36) | 1.54 | <.001 | .016 |
| 2. Perceived Emotional Synchrony | 5.50 (1.60)b | 5.04 (1.79)a | 5.55 (2.25) | 11.06 | <.001 | .014 |  | 5.86 (1.16)b | 5.03 (1.42)a | 5.11 (1.56)a | 16.60 | <.001 | .025 |
| 3. Intense Positive Emotions | 4.37 (1.98)b | 3.69 (2.02)a | 5.11 (2.00)b | 18.29 | <.001 | .022 |  | 6.05 (1.10)b | 5.06 (1.54)a | 5.54 (1.62) | 21.24 | <.001 | .032 |
| 4. Self-transcendent Emotions | 4.81 (1.96)b | 4.11 (2.11)a | 5.25 (2.20) | 18.09 | <.001 | .022 |  | 6.21 (.99)b | 5.48 (1.31)a | 5.45 (1.63)a | 19.00 | <.001 | .029 |
| 5. Self-transcendent Experience | 4.76 (1.86)b | 4.13 (1.95)a | 5.12 (2.21) | 16.54 | <.001 | .020 |  | 5.92 (1.20)b | 5.25 (1.33)a | 5.50 (1.43) | 9.15 | <.001 | .014 |
| 6. Situated social identity | 4.42 (2.10)b | 3.62 (2.08)a | 4.80 (2.39) | 21.30 | <.001 | .026 |  | 6.01 (1.16)b | 5.26 (1.41)a | 5.43 (1.53)a | 12.77 | <.001 | .020 |
| 7. Identity Fusion demonstration’s | 3.04 (1.34)b | 2.61 (1.27)a | 3.00 (1.51) | 14.58 | <.001 | .018 |  | 4.17 (.96)b | 3.24 (1.14)a | 3.50 (1.29)a | 27.67 | <.001 | .042 |
| 8. Identity Fusion Feminist | 3.15 (1.38)b | 2.54 (1.26)a | 3.73 (1.58)b | 31.44 | <.001 | .038 |  | 4.21 (.95)b | 3.55 (1.05)a | 4.00 (1.32) | 11.97 | <.001 | .019 |
| 9. Solidarity with Women | 5.85 (1.46)b | 5.56 (1.47)a | 5.71 (2.02) | 5.66 | .004 | .007 |  | 6.57 (.76)b | 6.25 (.91)a | 6.35 (1.00) | 4.68 | .009 | .008 |
| 1. Identity Fusion Women | 4.04 (1.05)b | 3.49 (.98)a | 3.93 (1.28) | 4.43 | <.001 | .048 |  | 4.20 (.87)b | 3.55 (.97)a | 3.67 (1.24)a | 17.28 | <.001 | .027 |
| 11. Collective Efficacy | 5.69 (1.48) | 5.52 (1.66) | 5.80 (1.98) | 1.92 | .147 | .002 |  | 6.32 (.91) | 6.06 (.95) | 6.16 (1.12) | 2.12 | .121 | .004 |
| 12. Positive Individual Growth | 3.80 (1.68)b | 3.22 (1.75)a | 3.62 (2.07) | 16.71 | <.001 | .020 |  | 4.57 (1.38) | 4.12 (1.55) | 4.42 (1.56) | 2.48 | .084 | .004 |
| 13. Positive Collective Growth | 4.37 (1.48)b | 3.84 (1.68)a | 4.20 (2.08) | 17.44 | <.001 | .021 |  | 5.21 (.98)b | 4.72 (1.22)a | 5.14 (.63) | 17.44 | <.001 | .021 |
| 14. Pro-women behavior | 3.13 (1.22)b | 2.55 (1.24)a | 3.43 (1.46) | 32.41 | <.001 | .039 |  | 4.09 (.84)b | 3.72 (.85)a | 4.27 (.80)b | 5.15 | .006 | .009 |

*Note.* Different superscripts represent significant differences (at least p < .05) conducted as post-hoc DMS tests. n(Demonstrators) = 1271; n(Non-Demonstrators) = 1583; n(Female) = 2393; n(Male)= 423; n(Non-Binary)= 38, after conducting post-hoc DMS tests. Age and political positioning scale have been controlled.
