# Supplementary material for: Collective Effervescence, Self-Transcendence, and Gender Differences in Social Well-Being During 8 March Demonstrations
Source: Front Psychol. 2020 Dec 11;11:607538. doi: 10.3389/fpsyg.2020.607538 (PMC7759529; doi:10.3389/fpsyg.2020.607538)
Supplement: Supplementary file 6 [file Table_6.DOCX]

**Table VI.**

*Regression analysis for the outcome variables*

| Dependent Variables | PES | | | Participation in demonstrations | | | Gender | | | | | Age | | | | | | Political Position | | | | | | |  | | |  | | |  |  |
| --- | --- | --- | --- | --- | --- | --- | --- | --- | --- | --- | --- | --- | --- | --- | --- | --- | --- | --- | --- | --- | --- | --- | --- | --- | --- | --- | --- | --- | --- | --- | --- | --- |
|  | *b* | *95% IC [LL, LU]* | *Beta* | *b* | *95% IC [LL, LU]* | *Beta* | *b* | *95% IC [LL, LU]* | | *Beta* | | *b* | | *95% IC [LL, LU]* | | *Beta* | | | *b* | | *95% IC [LL, LU]* | | *Beta* | | | *R* | | | *R^2^* | | |  |
| Self-Transcendence Experience | 0.76^**^ | [0.73, 0.79] | .64 | 10.00^**^ | [0.90, 10.09] | .28 | -0.13^*^ | | [-0.24, -0.20] | | -.03 | | -0.006^**^ | | [-0.009, -0.002] | | -.005 | | | -0.06^**^ | | [-0.09, -0.04] | | -.06 | | | .74 | | | .55^**^ | | |
| Situated Social Identity | 0.75^**^ | [0.72, 0.79] | .57 | 10.46^**^ | [10.35, 10.57] | .36 | -0.27^**^ | | [-0.39, -0.14] | | -.06 | | 0.006^**^ | | [0.002, 0.011] | | .04 | | | -0.12^**^ | | [-0.15, -0.09] | | -.10 | | | .72 | | | .52^**^ | | |
| Identity fusion’s demonstrators | 0.36^**^ | [0.33, 0.39] | .40 | 10.02^**^ | [0.09, 10.11] | .38 | -0.25^**^ | | [-0.35, -0.15] | | -.08 | | 0.001 | | [-0.003, 0.004] | | .001 | | | -0.05^**^ | | [-0.08, -0.03] | | -.07 | | | .61 | | | .37^**^ | | |
| Identity Fusion Feminist’s | 0.33^**^ | 0.30, 0.36] | .37 | 10.04^**^ | [0.09, 10.13] | .38 | -0.28^**^ | | [-0.38, -0.18] | | -.09 | | 0.004^*^ | | [-0.38, -0.18] | | -.04 | | | -0.06^**^ | | [-0.09, -0.04] | | -..08 | | | .58 | | | .34^**^ | | |
| Solidarity with Women | 0.36^**^ | [0.33, 0.39] | .42 | 00.62^**^ | [0.53, 0.72] | .05 | -0.08 | | [-0.19, 0.02] | | -.03 | | 0.005^*^ | | [0.01, -0.07] | | .04 | | | -0.04^**^ | | [-0.07, -0.02] | | -.06 | | | .51 | | | .26^**^ | | |
| Identity Fusion Women | 0.13^**^ | [0.11, 0.16] | .20 | 0.15^*^ | [0.07, 0.23] | .07 | -0.39^**^ | | [-0.48, -0.30] | | -.16 | | -0.001 | | [-0.002, 0.004] | | -.01 | | | -0.01 | | [-0.04, -0.01] | | -.02 | | | .29 | | | .09^**^ | | |
| Collective Efficacy | 0.35^**^ | [0.32, 0.38] | .39 | 0.58^**^ | [0.48, 0.67] | .21 | 0.03 | | [-0.09, 0.14] | | .01 | | -0.003 | | [-0.01, 0.00] | | -.09 | | | -0.07^**^ | | [-0.10, -0.04] | | -.09 | | | .47 | | | .22^**^ | | |
| Positive Individual Growth | 0.44^**^ | [0.41, 0.48] | .40 | 0.75^**^ | [0.64, 0.87] | .22 | -0.21^*^ | | [-0.35, -0.08] | | -.05 | | -0.012^**^ | | [-0.02, -0.01] | | -.08 | | | -0.09^*^ | | [-0.12, -0.06] | | -.09 | | | .51 | | | .26^**^ | | |
| Positive Collective Growth | 0.44^**^ | [0.41, 0.47] | .47 | 0.77^**^ | [0.67, 0.86] | .26 | -0.20^**^ | | [-0.31, -0.10] | | -.06 | | -0.01^*^ | | [-0.012, -0.005] | | -.07 | | | -0.08^**^ | | [-0.10, -0.05] | | -.09 | | | .59 | | | .34^**^ | | |
| Pro-women behavior | 0.32^**^ | [0.29, 0.34] | .39 | 10.01^**^ | [0.93, 10.10] | .41 | -0.21^**^ | | [-0.30, -0.12] | | -.07 | | -0.005^**^ | | [-0.008, -0.002] | | -.05 | | | -0.10^**^ | | [-0.12, -0.08] | | -.14 | | | .61 | | | .37^**^ | | |

*Note.* A significant *b* weight indicates the *beta* weight correlation are also significant.  *b* represents unstandardized regression weights. *Beta* indicates the standardized regression weights. LL and UL indicate the lower and upper limits of a confidence interval, respectively. Participants (0 = *non-demonstrator/followers/audience*, 1 = *demonstrator*), gender (1 = *female*, 2 = *male,* 3 = *non-binary*), political position (0 = *no position*, 1 = *extreme left* to 7 = *extreme right*),* indicates *p* <.05. ** indicates *p* <.01.
