# Supplementary material for: Collective Effervescence, Self-Transcendence, and Gender Differences in Social Well-Being During 8 March Demonstrations
Source: Front Psychol. 2020 Dec 11;11:607538. doi: 10.3389/fpsyg.2020.607538 (PMC7759529; doi:10.3389/fpsyg.2020.607538)
Supplement: Supplementary file 7 [file Table_7.DOCX]

**Table VII.**

*Brief Ethnographic description 8M-2020 Demonstrations by country.*

| **Country** | **Ethnographic description** | **Synchronized emotional expression** | **Context of repression** |
| --- | --- | --- | --- |
| Argentina | Collective protest actions, as well as massive political gatherings in public places, are integral to Argentina's political culture. In the case of the demonstration on March 8, under the slogan "not one less" the rejection of gender violence is expressed. In 2020, the most prominent chant was "The Rapist Is You". As is traditional in Argentine marches, the rejection of the budget adjustment and the precariousness of labor was also expressed. The 2020 demonstration had moderately less support than that of 2019, presumably because of the pro-abortion identification that currently prevails in the movement, with which not all Argentine feminists identify. A large part of the scarves, banners and other symbols used in 2019 was violet, representing the feminist struggle, while in 2020, the color green, typical of the pro-abortion movement, stood out. | - choreography "Un violador en tu camino" (A rapist in your way) - Manifesto reading - Synchronized singing - Performances - Mask-covered faces | No violence is reported during demonstrations or repressive actions by the security forces. |
| Brazil | March 8, 2020, a rainy day, was marked by protests in the streets of Salvador for more rights. Several feminist collectives, trade union movements, and leftist political parties that brought together women of all ages walked along the edge of the beach with the slogan "Women against Bolsonaro, for our lives, democracy, rights and justice for Marielle (councilwoman murdered in Rio de Janeiro) January 14, 2018) and Dandaras (transvestite murdered in 2017)". They also protested against the increase in femicides, the dismantling of Social Security, and the negligence of the Bolsonaro government. Amid the demonstrations and shouts of "Mariele lives", outside Bolsonaro and not him, banners and posters acquire a political tone, with criticism of the government of Jair Bolsonaro, women for democracy, against fascism, patriarchy, the defense of women's rights, the legalization of abortion, for the end of racism and prejudice against LGBT people. Many of the women who participated in the march wore feminist collective T-shirts and protest stickers. A group of young people linked to the popular youth uprising accompanied the walk with percussion, performance, and protest songs. | - choreography "Un violador en tu camino" (A rapist in your way) - Synchronized singing - Performances - Batucada | Besides some isolated events, there was no violence or repressive actions during 8M |
| Chile | In Chile, specifically in Antofagasta, the 8M demonstrations were massive but mostly separatist. In them, music was a central element; chants, choreographies related to the Chilean-born project "Un violador en tu camino" (A rapist in your way) were observed. The people used colors that were most representative of feminist struggles, but also costumes and banners. On this occasion, people and collectives who had never before attended the 8M demonstration were observed. In general, women emphasized gender violence and harassment, but they also mentioned the body as a space for liberation from social mandates. The recurrent slogans referred to sexual and reproductive rights and violence against women. The level of emotional intensity was high, and accompanied by symbolic and artistic expressions. | - choreography "Un violador en tu camino" (A rapist in your way) - Synchronized singing - Performances - Mask-covered faces | In general, there were events of police violence in some cities during the demonstrations. However, in most cities, these events were limited, and the demonstrations were conducted peacefully. |
| Colombia | The commemoration of 8M in Colombia takes place in a context of large-scale social mobilizations that began in September 2019 and included strikes, blockades of highways, and a strong repressive reaction by the police. Student organizations and indigenous movements supported this demonstration. Commemorative events were conducted during the previous days (March 6 and 7) and a massive demonstration in the main cities of the country that included the performance of the project "Un violador en tu camino" (a rapist in your way), graffiti, dances, songs and the dissemination of reports about gender violence. In general, large groups of women, activists, and feminists attended the demonstration. There was a large presence of female university students. In some cities such as Barranquilla (one of the cities where the sample was collected), the demonstrations lasted until the next day with academic activities and protests in government buildings, demanding justice for the femicide that had occurred in the country. | - choreography "Un violador en tu camino" (A rapist in your way) - Synchronized singing - Batucada - Performances - Mask-covered faces | Despite the agitating social climate of the pre-March 8 mobilizations, the Women's Day demonstrations did not include acts of police repression or violence, except in occasional cases of confrontations between a few small groups and the police. |
| Ecuador | In Ecuador, especially in Quito, a massive demonstration brought together women of all ages mainly to demand the protection of their rights and to denounce the femicides that had occurred in previous months. The demonstration was attended by groups of native women, who denounced the inequality as women and as native people. At different times during the demonstration, there were performances and artistic demonstrations that included the Chilean choreography "Un violador en tu camino" (A rapist in your way) and mask in allusion to the artistic work "La ciudad de las mujeres innobles" (The city of ignoble women), by Rosa Amelia Poveda, which reflects on gender violence. | - choreography "Un violador en tu camino" (A rapist in your way) - Performances - Twerking | There was no repression or violence against the women participating in the demonstration by the security forces. |
| Spain | In Spain, the 8M demonstrations were massive and mostly by women, although not separatists. In different cities, from previous days and the same day of the demonstration, the choreography of "(Un violador en tu camino) a rapist on a road" was carried out several times. During the demonstration, the colors purple and green prevail in the assistants, who go organizing by groups supporting different causes (Against gender violence, racism, migration, pro-abortion). Also present were the batucada, as every year, as well as cultural expressions, performances, and songs. | - choreography "Un violador en tu camino" (A rapist in your way) - Batucada - Human chain - Synchronized singing | No events of violence or repression were observed during the demonstrations. |
| México | In general, the 8M demonstration was massive and had great national exposure. In different cities of Mexico, it was possible to observe expressions of protest and repudiation by women tired of the gender-based aggressions that result in a serious problem of femicide of at least 10 women every day in the country. Family members and friends of the victims were present at the demonstrations demanding justice. The mobilizations were emotional and full of symbolism, including choreography, body movements, shouts, etc., since many of them reached memorials dedicated to the murdered women. There were also minutes of silence and large bonfires with flags remembering the feminist struggles and gender violence. In some cities such as Coahuila (one of the cities where the sample was collected), the 8M demonstration was massive for the first time and separatist (only women in the demonstration). The demonstration, and the women's general strike the following day, was felt strongly and consolidated the visibility of a profound gender problem in the country. | - choreography "Un violador en tu camino" (A rapist in your way) - Ceremony in memory of victims - Synchronized singing - Batucada - Mask-covered faces | There were small physical confrontations with opposing organizations ( pro-life). There was violence towards stores, monuments, offices, and police. |
| Peru | The demonstrators, mostly women, and from LGTBIQ+ collectives, are mobilizing along the most important highways in the center of the capital city and other cities. In addition to banners and chants alluding to the struggle for women's rights, banners and chants are usually prepared for the event, as well as costumes and makeup that recall milestones in this movement, as well as historical events that have given strength to the collectives' demands, some of which are the forced sterilizations or disappearances and emblematic femicides. The protesters generally organize in groups that defend certain agendas with greater emphasis (the feminist struggle, gender violence, the rights of the transgender collective, etc). The event usually combines the space of protest and claiming with the use of artistic expressions, mainly music and performance. The colors purple and green stand out in the clothing and paraphernalia, as well as black and red as a sign of remembrance and mourning for femicide and gender violence in general. | - choreography "Un violador en tu camino" (A rapist in your way) - Batucada - Performance - Mask-covered faces | No events of repression or violence by police forces were observed during the 8M demonstrations. |
| Portugal | The 2020 Women's Day, in Portugal, was marked with demonstrations all over the country and by a feminist strike. The feminist organization “Rede de Março” (March Network; a national platform that brings together collectives, associations, political organizations, unions and individual people), promoted the national feminist strike (called for the second consecutive year), and organized simultaneous protests and demonstrations, in the cities of Amarante, Aveiro, Braga, Coimbra, Évora, Faro, Lisbon, Porto, Viseu, Vila Real and Ponta Delgada. In parallel, the Democratic Women's Movement (MDM) organized a single event in the Portuguese capital, Lisbon that included a march and a concentration, calling women from all over the country, from north to south. In both initiatives attended both women and men, national and foreign citizens, coming together for a common goal. The initiatives were marked essentially by demands against gender discrimination, gender inequality and gender-based violence that persists in the streets, in schools, and in the workplace. The events also drew attention to the scourge of domestic violence, and to the need for more preventive and combat policies and actions against it. The events were colored mostly by purple; songs, speeches, and shouts of protesters filled participants held posters and banners, and the environment of the events. | - choreography "Un violador en tu camino" (A rapist in your way) - Batucada - Performance | There was no repression of any kind during the demonstrations. |
