# Supplementary material for: Collective Effervescence, Self-Transcendence, and Gender Differences in Social Well-Being During 8 March Demonstrations
Source: Front Psychol. 2020 Dec 11;11:607538. doi: 10.3389/fpsyg.2020.607538 (PMC7759529; doi:10.3389/fpsyg.2020.607538)
Supplement: Supplementary file 8 [file Table_8.DOCX]

**Table VIII.**

*Data Collections Strategy 8M-2020 studies by Country.*

| **Country** | **Data Collection Strategy** |
| --- | --- |
| Argentina | The data collection was done for convenience. The survey was shared via a link to the Qualtrics application that was distributed, after a brief explanation of the study, to university students who reported attending the demonstration. They also shared the link with acquaintances who participated in the march. In addition, the link was shared via social media, e-mails, and email to participants and people who had followed the demonstrations through the media and social networks (non-demonstrators, supporters). The data was collected between March 8 and March 13, 2020 and the approximate time to complete the survey was 30 minutes. |
| Brazil | The data collection was done for convenience. The survey was shared through social networks, emails and electronic media to participants and people who had followed the demonstrations through the media and social networks (Non-protesters, supporters). The data was collected between March 8 and March 17, 2020 and the approximate time to complete the survey was 30 minutes. |
| Chile | Data collection was conducted for convenience. The survey was shared through a link on social media, among students of the Universidad Católica de Chile, friends, and colleagues (snowball sampling). The data was collected between March 8 and 20, 2020, and the approximate time to complete the survey was 25 minutes. |
| Colombia | The data collection was conducted for convenience. The survey was shared by providing a link and a QR code to the Qualtrics application that was distributed during the demonstrations to participants and interested audience members after a brief explanation of the study. The link was also shared via email to social psychology students and they were asked to share the link with their friends and family. The data was collected from March 8th to 15th and the approximate time to complete it was 30 minutes. |
| Ecuador | The data was collected between March 8 and 12 through an online link sent for convenience to members of groups and organizations close to the subject, as well as through social media to the general population. In both cases, e-mail and WhatsApp were also used. |
| Spain | The data collection was done for convenience. The survey was shared through a link and a QR code of the qualtrics application that was distributed during the demonstrations to interested attendees after a brief explanation of the study. Also, the link of responses was shared through social networks, emails and electronic media to participants and people who had followed the demonstrations through the media and social networks (Non-protesters, followers). The data was collected between March 8 and March 13, 2020 and the approximate time to complete the survey was 30 minutes. |
| Mexico | In Mexico, the survey was done with a procedure of convenience, which consisted of creating a micro-site where the purpose of the study, the treatment and safeguard of the data, a brief summary of the informed consent, and the assertion of the scientific (and non-remunerated) use of the participants were explained. The micro-site had a short link that was shared by social networks (personal and laboratory pages) on FB, Twitter accounts, and in the present and past groups of online classrooms. Also, it was sent to a mailing list. In all cases people were asked to respond to the survey, and also to share it with their own contacts. |
| Peru | The data was collected between March 8 and 10 through an online link sent for convenience to members of groups and organizations close to the subject, as well as through social media to the general population. In both cases, e-mail and WhatsApp were also used. |
| Portugal | The data collection was done for convenience. The survey was shared through a Link and a QR code from the qualtrics application that was shared to the general population, resident in Portugal, through Facebook and Instagram Ads, Facebook groups, and Whatsapp. The data was collected between March 8 and 13, 2020 and the approximate time to complete the survey was 30 minutes. |
